# Translational enhancement of target endogenous mRNA in mammalian cells using programmable RNA-binding pentatricopeptide repeat proteins

**DOI:** 10.1038/s41598-023-50776-z

**Published:** 2024-01-02

**Authors:** Ning Ping, Sayuri Hara-Kuge, Yusuke Yagi, Tomohiko Kazama, Takahiro Nakamura

**Affiliations:** 1 Faculty of Agriculture, Kyushu University, Fukuoka, 812-8581 Japan; 2 EditForce, Inc., Fukuoka, 819-0395 Japan

**Keywords:** Expression systems, Translation, Biotechnology, Molecular biology, Gene regulation

## Abstract

Programmable protein scaffolds are invaluable in the development of genome engineering tools. The pentatricopeptide repeat (PPR) protein is an attractive platform for RNA manipulation because of its programmable RNA-binding selectivity, which is determined by the combination of amino acid species at three specific sites in the PPR motif. Translation is a key RNA regulatory step that determines the final gene expression level and is involved in various human diseases. In this study, designer PPR protein was used to develop a translational enhancement technique by fusion with the translation initiation factor eIF4G. The results showed that the PPR-eIF4G fusion protein could activate the translation of endogenous *c-Myc* and *p53* mRNAs and control cell fate, indicating that PPR-based translational enhancement is a versatile technique applicable to various endogenous mRNAs in mammalian cells. In addition, the translational enhancement was dependent on both the target position and presence of eIF4G, suggesting the presence of an unknown translation activation mechanism.

## Introduction

Over the last two decades, genome and transcriptome research has led to considerable progress in molecular biology and applied fields, especially in the development of genome editing techniques such as zinc finger nuclease (ZFN), transcription activator-like effector nuclease (TALEN), and clustered regularly interspaced short palindromic repeat (CRISPR) systems^1,2^. CRISPR/CRISPR-associated protein 9 (Cas9) has been widely applied via combining gRNA (CRISPR RNA, trans-activating CRISPR RNA) with Cas9 proteins using homologous recombination and non-homologous end joining to achieve genome editing, including the removal and insertion of DNA sequences and base substitutions^3–5^.

As the understanding and manipulation of DNA have become prevalent, the importance of RNA functions has also increased. Large-scale analysis of RNAs associated with genome projects has revealed the great variety of alternative splicing events that occur in the transcriptome, and large amounts of non-coding RNAs have been discovered^6,7^. In addition, the potential risk of off-target effects that irreversibly change genomic DNA has increased in recent studies using genome-editing techniques. Therefore, the development of elaborate and versatile RNA manipulation techniques is anticipated to enable the reversible control of the expression of genetic information^8^.

Available RNA manipulation techniques are classified as nucleotide- or protein-based systems. Representative nucleotide-based techniques include the use of antisense oligonucleotides (ASOs) and miRNAs/siRNAs based on RNA interference (RNAi). Several studies have demonstrated their utility in basic biological research and medical applications^9,10^. Although these can target specific RNA sequences, they can only be used for the suppression of target RNA levels or functions and require the coordinated action of intracellular factors^11,12^. Recently, several studies have shown that Cas13a, an RNA-targeting CRISPR/Cas system, can be used for various RNA manipulation techniques, including knockdown, visualization, splicing control, translation activation, and RNA base substitution by fusion with additional functional domains^13–16^. The Cas13a technique is more elaborate than ASO or siRNA, however, base-to-base interactions can easily cause off-targeting effects, as shown by RNAi and DNA targeting using the CRISPR/Cas9 system. Protein-based tools have potential advantages, as seen with the various RNA-binding protein factors that are responsible for a wide range of cellular RNA processing reactions. Various attempts have been made to use an RNA-binding protein fused with an additional functional domain to target and control a single target RNA in living cells^17^. The use of MS2 phage coat protein is a traditional example. The MS2 protein, derived from bacteriophages, recognizes specific sequences and structures of the 19 nt of translational operator RNA (TR). Based on these characteristics, MS2-based systems have been used for RNA visualization, RNA degradation, and translation activation^18,19^. However, MS2-based systems cannot be applied to most endogenous RNA, because the RNA sequence recognition ability of MS2 cannot be modified. This limiting trait was partially improved by the appearance of Pumilio/fem-3 mRNA-binding factor (PUF). The PUF protein consists of 8–9 repeat PUF motifs. In the RNA-binding, a single PUF motif corresponds to one nucleotide, and RNA base specificity is largely determined by the amino acid species at two particular positions of the PUF motif^20,21^. Based on this knowledge, various RNA manipulation tools including translational enhancement, degradation, and RNA visualization have been developed using PUF proteins^22–24^. However, the PUF protein-based system has a significant limitation in that RNA sequence recognition is not fully programmable, and the recognized target length cannot be increased to more than 8 or 9 bases.

The limitations of protein-based techniques can be potentially resolved using pentatricopeptide repeat (PPR) proteins, whose gene families are extraordinarily expanded only in land plants^25^. PPR proteins are sequence-specific RNA-binding proteins involved in RNA processing, including RNA cleavage, mRNA stabilization, translation, and RNA editing in a gene-specific manner^26^. In contrast to the PUF protein, the PPR protein comprises variable repetitions of PPR motifs (2–27). The sequence-specific RNA-binding mechanism of the PPR protein has been elucidated—the number of PPR motifs in a PPR protein determines the target RNA length, with one PPR motif corresponding to one nucleotide; the recognized RNA base was determined by the amino acid at positions 2, 5, and 35 within the PPR motif^27,28^. These programmable RNA-binding characteristics provide attractive engineering platforms for sequence-specific designer RNA-binding proteins. With improvements in PPR technology, dozens of designer PPR proteins (18 nt RNA recognition) have been shown to solely interact with the respective target RNAs and their application in the splicing of endogenous mRNA in cultured animal cells has been demonstrated^29^. Designer PPR proteins with various modes of action are expected to be fused with additional protein domains. We previously performed translational enhancement using a reporter gene assay in cultured animal cells with a natural PPR protein, CRR4, fused with the translation initiation factor eIF4G^30^.

In the present study, we expanded the development of designer PPR proteins as a versatile translation enhancement technique by investigating their applicability in targeting endogenous mRNAs using designer PPR proteins fused to eIF4G. *p53* and *c-Myc* mRNAs were chosen as target molecules because overexpression of p53 and c-Myc inhibits and promotes cell proliferation, respectively; therefore, the translational activation of *p53* or *c-Myc* mRNA can be evaluated by changes in cell proliferation. We showed that, with the expression of the PPR-eIF4G fusion gene, the translational enhancement of p53 inhibited cell growth, whereas that of c-Myc promoted cell growth with no change in their RNA levels. The translational enhancement was dependent on both the target position and the presence of eIF4G, suggesting the presence of a new translation activation mechanism. This study indicates that the PPR-based translational enhancement technique is a versatile system that can enhance the translation of endogenous mRNAs and control cell fate.

## Results

### Translational activation of *p53* mRNA using designer PPR proteins

We previously showed that a PPR-based target-specific translational activation system using a natural PPR protein fused with the truncated eIF4G (607–1600 aa) enabled translational enhancement in an artificial reporter gene assay^30^. In this study, we attempted to expand this application by targeting endogenous RNA using a designer PPR protein that recognizes desirable target RNA sequences^29^. We first selected *p53* as a model mRNA because it has a short 5′ UTR (63 nt) and elevation of p53 protein levels is expected to inhibit cell growth.

**Figure 1:**
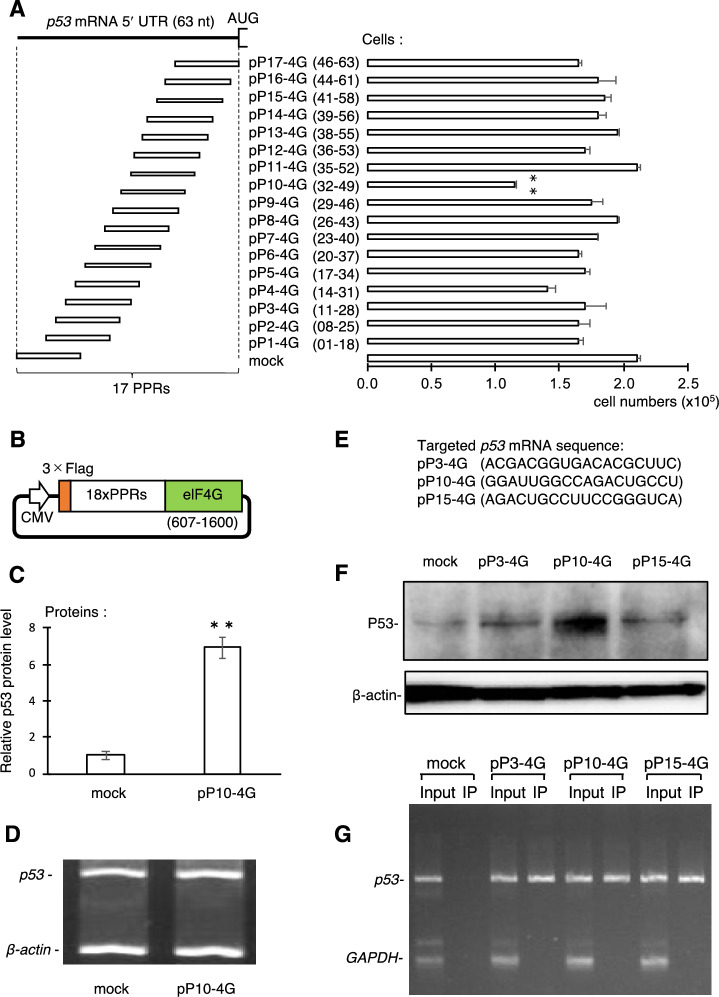
Translational enhancement of *p53* mRNA by pentatricopeptide repeat (PPR)-eIF4G fusion protein. **(A)** *p53* mRNA 5′ UTR and target position of designed PPR-eIF4G fusion gene (pP1-4G to pP17-4G). Number of Hela cells was determined 2 d after transfection with designed PPR-eIF4G fusion genes. Mock indicates transfection with empty vector. Unpaired two-tailed Student’s *t*-test, *N* = 3, **p < 0.01. Error bars indicate standard deviation. **(B)** Structure of PPR-eIF4G fusion gene plasmid. The PPR protein gene with 18 PPR motifs (i.e., 18 nt recognition) was fused with 3 × FLAG and truncated eIF4G (607–1600 aa), and integrated in expression plasmid under the CMV promoter. **(C)** p53 protein level analyzed by ELISA with or without pP10-4G transfection. **(D)** RT-PCR analysis for *p53* mRNA with or without pP10-4G transfection. *β-actin* was used as an internal control. **(E)** Target RNA sequence for PPR-eIF4G fusion gene (pP3-4G, pP10-4G, and pP15-4G). **(F)** Western blot analysis to examine p53 protein levels in absence (mock) or in presence of pP3-4G, pP10-4G, and pP15-4G. **(G)** RNA–protein co-immunoprecipitation and RT-qPCR (RIP-PCR) analysis of interaction between PPR-eIF4G fusion protein and targeted *p53* mRNA in cultured cells. The RNA from total lysate (input) or coprecipitated RNA (IP) with PPR-eIF4G fusion protein (pP3-4G, pP10-4G, and pP15-4G) was used for RT-PCR analysis. *GAPDH* was used as an internal control.

We prepared 17 designer PPR proteins with 18 PPR motifs, i.e., 18 nt recognition, for 63 nt of the 5′ UTR of *p53* mRNA with a 16 nt overlap (Fig. 1A and Supplemental Table 1). The designer PPR gene was fused to 3 × FLAG and eIF4G genes at the N- and C-termini, respectively, under the control of a CMV promoter (Fig. 1B). The p53-targeting PPR-eIF4G fusion gene, designated pP1-4G to pP17-4G, was transiently transfected into HeLa cells and its effect was first screened by changes in cell growth 48 h after transfection. Significant inhibition of cell growth was observed when the pP10-4G construct was introduced (Fig. 1A), suggesting the translational enhancement of *p53* mRNA.

ELISA revealed an approximately sevenfold accumulation of p53 protein in the presence of pP10-4G (Fig. 1C), while RT-PCR analysis showed that p53 RNA levels did not change under these conditions (Fig. 1D). The elevated accumulation of p53 protein in HeLa cells was confirmed by western blot analysis (Fig. 1F). The equivalent pP10-4G effect was also observed in Hek293 cells (Figure S1A). These results showed that the PPR-eIF4G fusion protein activated the translation of endogenous p53 mRNA without altering the amount of RNA in cultured human cells.

To eliminate the possibility of some PPR proteins not interacting with the target *p53* mRNA, RNA–protein co-immunoprecipitation and semi-quantitative RT-PCR (RIP-PCR) analyses were conducted using the plasmids pP3-4G, pP10-4G, and pP15-4G. The PPR-eIF4G protein was immunoprecipitated from the cell lysate using anti-FLAG tag beads. The expression of PPR-eI4FG fusion proteins were verified by western blot (Fig. S2A). RNA was extracted from the precipitated fraction, and the presence of *p53* mRNA was examined by RT-PCR, using *GAPDH* mRNA as a negative control. The RT-PCR product of *p53*, but not *GAPDH*, was observed in the precipitate from cultured cells transfected with pP3-4G, pP10-4G, or pP15-4G (Fig. 1G). PCR products from *p53* and *GAPDH* mRNAs appeared in the input sample but not in the precipitant prepared from non-transfected cells. This indicated that all PPR-eIF4G fusion proteins (pP3, pP10, and pP15) specifically interacted with the target *p53* mRNA in cultured cells, suggesting that simple binding of the PPR-eIF4G fusion protein is not sufficient for the translational activation.

**Figure 2:**
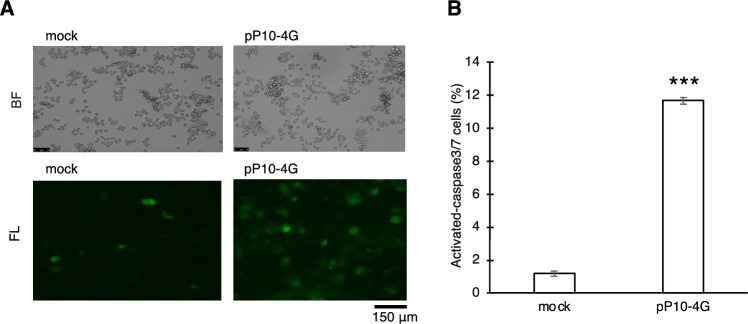
Translational enhancement of *p53* mRNA by PPR-eIF4G fusion gene induces apoptosis. **(A)** Apoptosis activity was analyzed using a florescent probe recognizing activated caspase-3/7, with or without pP10-4G transfection. Representative microscopic bright field (BF) and fluorescent (FL) images are shown. Mock indicates transfection with the empty vector. **(B)** Estimation of the ratio of fluorescence-positive cells by flow cytometry. Unpaired two-tailed Student’s *t*-test, *N* = 3, ***p < 0.001. Error bar indicates the standard deviation.

The p53 protein is a known a transcription factor whose activation stimulates caspase-3/7 followed by apoptosis, normally due to stress and/or DNA damage^31,32^. The inhibition of cell growth by PPR10-eIF4G may be due to the induction of the p53 signal transduction pathway. To confirm this hypothesis, apoptotic activity was examined with and without PPR10-eIF4G transfection. PPR10-eIF4G transfected Hela cells were analyzed by microscopy using a fluorescent probe recognizing activated caspase-3/7, a typical apoptosis marker. We observed a significant increase in the number of fluorescence-positive cells (Fig. 2A), which corresponded to a tenfold increase, based on flow cytometry (Fig. 2B), indicating that translational activation of *p53* mRNA by PPR10-eIF4G induces apoptosis and inhibits cell growth.

**Figure 3:**
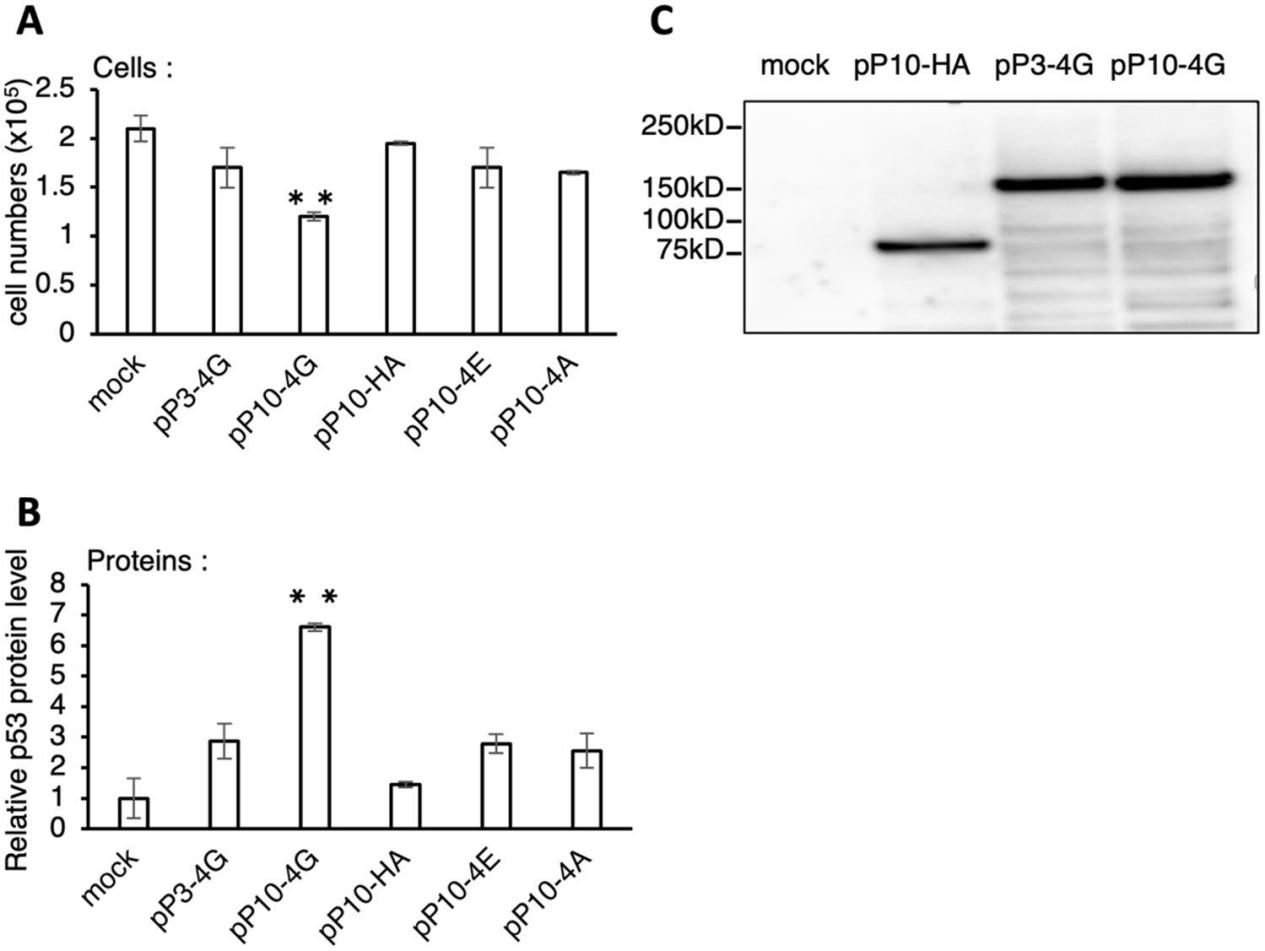
Role of PPR and eIF4G domain in the translational enhancement. The translational enhancement was evaluated by transfection with pP10-4G, pP10-HA, pP10-4E, pP10-4A, and pP3-4G. **(A)** Cell number evaluated as shown in Fig. 1. Unpaired two-tailed Student’s *t*-test, *N* = 3, **p < 0.01. Error bar indicates the standard deviation. **(B)** Analysis of protein accumulation level, same as in (A). **(C)** Protein level with mock, pP10-HA, pP3-4G, or pP10-4G transfection examined by western blot analysis using anti-FLAG antibody.

To gain mechanistic insights into the role of pP10-4G, the contributions of the PPR and eIF4G moieties to the translational enhancement were examined using a PPR-HA gene, which contains an HA tag instead of an eIF4G domain. Transfection analysis showed that suppression of cell growth was observed only with the addition of pP10-4G and not pP10-HA or pP3-4G (Fig. 3A). Additionally, a significant increase in p53 protein levels (up to sevenfold) was observed only in the presence of pP10-4G (Fig. 3B). pP10-4G, pP10-HA, and pP3-4G protein levels were similar under these conditions (Fig. 3C). During translation initiation, eIF4G cooperates with eIF4E, a cap-binding protein, and eIF4A, an RNA helicase. To evaluate the function of eIF4G in this system, PPR10-eIF4E and PPR10-eIF4A fusion protein genes were constructed (designated pP10-4E and pP10-4A, respectively) and used in the transfection assay. pP10-4E and pP10-4A induced a slight inhibition of cell growth and an elevation of protein accumulation, but the difference was not significant (Fig. 3A,B). This indicated that both target recognition by PPR10 (position 32–49) and translational activation by eIF4G were indispensable for the translational enhancement of *p53* mRNA.

### Translational enhancement of *c-Myc* mRNA using designer PPR protein

**Figure 4:**
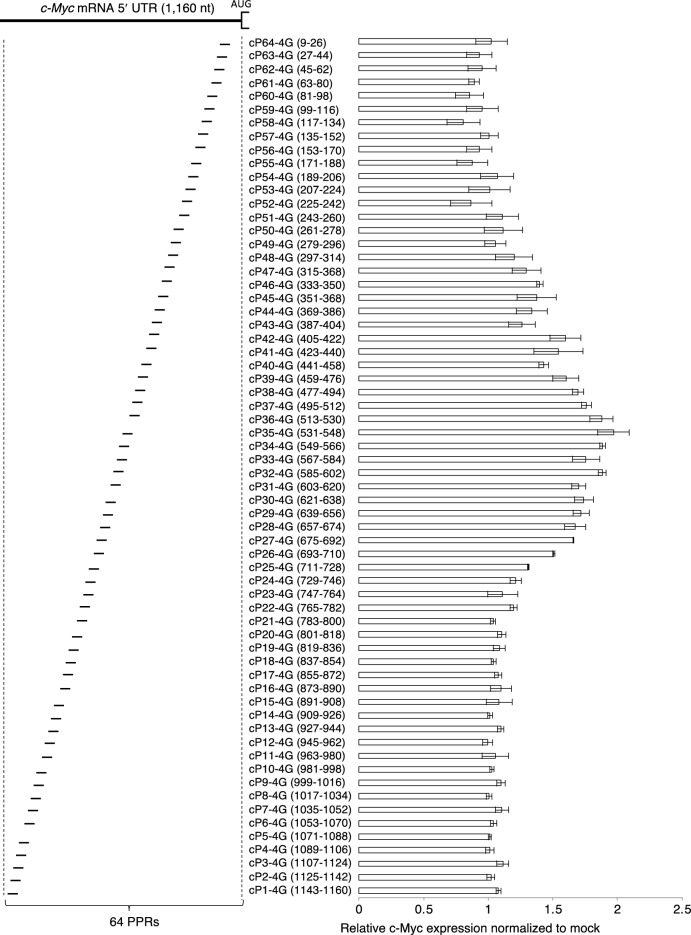
Screening for translational enhancement of *c-Myc* mRNA by the PPR-eIF4G fusion gene. c*-Myc* mRNA 5′ UTR and the target position of the designed PPR-eIF4G fusion gene (cP1-4G to cP64-4G); 64 PPR proteins of 18 nt recognition were designed to cover the 5′ UTR of *c-Myc* mRNA. c-Myc protein accumulation in HEK293 cells was analyzed by a homologous time-resolved fluorescence (HTRF) assay 2 d after transfection with the PPR-eIF4G fusion gene plasmids. Error bar indicates the standard deviation (N = 3).

We applied this technique to a second target mRNA, *c-Myc. c-Myc* mRNA contains a relatively long 5′ UTR (1,160 nt) compared to *p53*. We prepared 64 designer PPR proteins, i.e., 18 nt recognition, to cover the 5′ UTR of the *c-Myc* mRNA, which we fused with 3 × FLAG and the eIF4G gene (same as for the *p53* mRNA) and designated cP1-4G to cP64-4G (*c-Myc*-targeting PPR-eIF4G; Fig. 4). The plasmid containing the PPR-eIF4G gene was introduced into HEK293 cells, and the change in c-Myc protein levels was examined 48 h after transfection using a homologous time-resolved fluorescence (HTRF) assay that enables high-throughput quantification of protein levels. An increase in c-Myc protein levels was observed with the addition of multiple PPR-eIF4G plasmids designed to recognize the middle position (243–782) of the *c-Myc* mRNA (Fig. 4). The most pronounced effect, a twofold increase in c-Myc protein levels, was obtained by transfection with cP35-4G, which recognizes positions 531–548.

**Figure 5:**
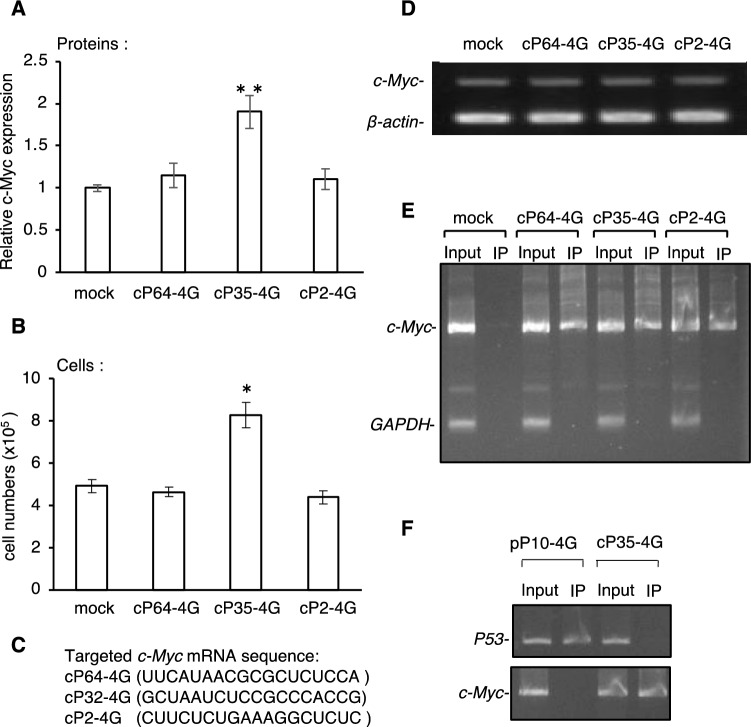
Evaluation of PPR-eIF4G fusion protein in the translational enhancement of *c-Myc* mRNA. **(A)** Effect of *c-Myc* targeting PPR-eIF4G fusion gene (cP64-4G, cP35-4G, or cP2-4G) on c-Myc protein level examined by HTRF assay in HEK293 cells 2 d after transfection. The results of statistical analysis are shown, N = 6, *p < 0.05, **p < 0.01. Error bar indicates the standard deviation. **(B)** the cell number examined using automated cell counters in HEK293 cells, same as in (A). **(C)** Target RNA sequence for PPR-eIF4G fusion gene (cP64-4G, pP32-4G, and pP2-4G). **(D)** *c-Myc* RNA level determined by RT-PCR using the transfected cells in (A). *β-actin* was used as an internal control. **(E)** RIP-PCR was conducted, as shown in Fig. 1E, to analyze the interaction between the PPR-eIF4G fusion protein (cP3-4G, cP10-4G, and pP15-4G) and the targeted *c-Myc* mRNA in cultured cells. Mock indicates transfection with the empty vector. **(F)** RIP-PCR analysis of the cross reactivity of *p53*-targeting pP10-4G and *c-Myc*-targeting cP35-4G by detection of *p53* and *c-Myc* mRNA.

The effect of cP35-4G on the translational enhancement was further analyzed using other PPR fusion genes, cP2-4G and cP64-4G, which recognize the positions 27–44 and 1,143–1,160, respectively. An up to twofold elevation in c-Myc protein levels was observed in the presence of cP35-4G but not in the presence of cP2-4G and cP64-4G by HTRF assay (Fig. 5A). The elevation of c-Myc protein level was verified by western blot analysis (Fig. S1B). Notably, the number of cells increased in the presence of cP35-4G (Fig. 5B). RT-PCR analyses revealed that endogenous *c-Myc* mRNA levels were not altered by transfection with any of the PPR-eIF4G fusion plasmids (Fig. 5D). In addition, both PPR and eIF4G moieties were required for the translational activation because the removal of eIF4G from cP35-4G did not elevate c-Myc protein levels, similar to that in the p53 experiment (Fig. S4).

The interaction of the respective PPR fusion proteins with the target RNA was analyzed by RIP-PCR, as performed for the *p53* experiment. The RIP-PCR analysis demonstrated that expression of all three PPR proteins (cP2-4G, cP35-4G, and cP64-4G) and interaction with the target *c-Myc* mRNA (Fig. 5E and Fig. S2B). We evaluated the cross-reactivity of the PPR fusion proteins that recognize *p53* and *c-Myc* mRNA. pP10-4G and cP35-4G enhanced the translation of *p53* and *c-Myc* RNA, respectively. When the RNA coprecipitated with *p53* targeting pP10-4G was subjected to RT-PCR analysis, only *p53* RNA was detected. In contrast, only *c-Myc* RNA was detected by RT-PCR using the RNA template coprecipitated with *c-Myc* targeting PPR of cP35-4G (Fig. 5F). This indicated that the designer PPR proteins interact solely with the target mRNAs, as intended. Collectively, these experiments showed that the PPR-eIF4G fusion gene can be applied to the translational enhancement of *c-Myc* mRNA and stimulate cell growth.

### Role of eIF4G domain in the translational enhancement

**Figure 6:**
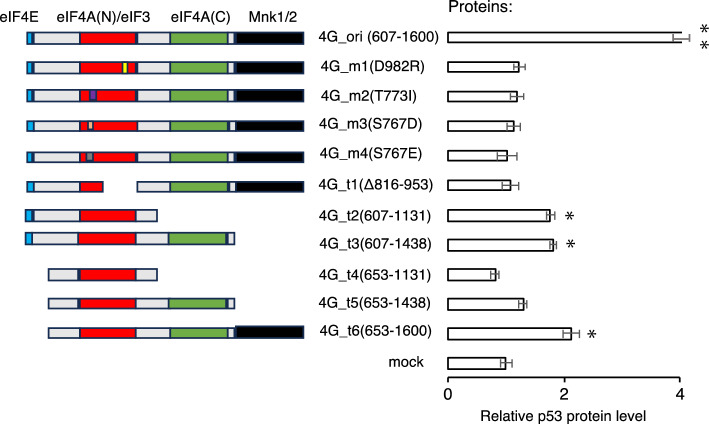
Evaluation of eIF4G domain in translational enhancement by PPR-eIF4G fusion gene. Schematic representation of mutated or truncated eIF4G with highlighted functional domains. Digit in parentheses indicate amino acid position. The translational enhancement efficiency was evaluated by analyzing p53 protein levels using ELISA as done in Fig. 1C. Unpaired two-tailed Student’s *t*-test; *N* = 5; **p < 0.01 and *p < 0.05.

A series of experiments showed that the translational enhancement was observed only when the PPR gene was fused with eIF4G, but not with eIF4A and 4E. To validate the involvement of the eIF4G moiety and gain mechanistic insights, we tested various truncated or mutated versions of eIF4G according to the functional region in eIF4G^43^ (Fig. 6). The truncated or mutated eIF4G was fused to pP10, which exhibited the most pronounced translational enhancement by targeting *p53* mRNA (Fig. 1). The translational enhancement, which was observed in the original eIF4G moiety (4G_ori, 607–1600 aa), was abolished by mutation or truncation of the eIF4A(N)/eIF3 interaction (4G_m1, m2, m3, m4, and t1). The PPR-eIF4G fusion genes that contain truncation of the eIF4E or Mnk 1/2 interaction region induced the statistically significant translational enhancement (1.7–2.0-fold), although the efficiency was lower than that of 4G_ori. These results suggest that interactions between various translational initiation factors are involved in the translational enhancement by PPR-eIF4G fusion protein.

## Discussion

Designer PPR proteins can achieve various modes of action by fusion with effector protein domains, such as splicing control, RNA editing, and translational enhancement. In this study, we expanded the utility of the translational enhancement technique from our previous study using a natural PPR protein and reporter gene, and showing that the designer PPR-eIF4G fusion protein can enhance the translation of endogenous *p53* and *c-Myc* mRNAs by up to sevenfold without changing the mRNA level, and control cell fate by inhibition and promotion of proliferation, respectively, in different cell lines. These results indicate that PPR-based translational enhancement is a versatile technique applicable to various endogenous mRNAs in cultured mammalian cells.

Artificial translational activation of endogenous mRNA has been achieved in few systems apart from PPR technology. SINEUP (also called RNAe)—a nucleotide-based translational enhancement technique—is an antisense long noncoding RNA in which an embedded SINE B2 element upregulates the translation of sequences partially overlapping with the target sense mRNA. This system increased the expression of endogenous mouse sex determining region Y-box 9 (SOX9) and human ubiquitin carboxy-terminal hydrolase L1 (UCHL1) proteins by up to 2.7- and 1.7-fold, respectively^33–35^. One limitation is that the artificial SINEUP sequence must be designed for a limited region near the translation initiation codon^35^. More recently, this traditional SINEUP system was greatly improved by coupling it with the RNA-targeting CRISPR/Cas13 system^36^. CRISPR13-SINEB2 was used to target *p53*, *c-Myc,* and *PTEN* mRNA and activate translation by up to 4.2 fold. The translational activation of *p53* and *c-Myc* mRNA induced apoptotic activity by 2.5-fold activation and 0.5-fold reduction, respectively, with comparable efficiency to the PPR system in the current study. Remarkably, CRISPR-SINEB2 has been shown to inhibit tumor development and metastasis in mice.

CRISPR/Cas13 has also been used for translational enhancement via fusion with a protein-based translational activation domain. CRISPR/Cas13 fused with the YTH N6-methyladenosine RNA-binding protein F1 (YTHDF1) fragment demonstrated a 1.4-fold translational activation by targeting a luciferase mRNA in HEK293T cells^16,37^. Another translational enhancement technique used in prokaryotic cells with initiation factor 3 (IF3) demonstrated a 2.6-fold upregulation of endogenous *LacZ* mRNA in *E. coli*^38^.

Despite recent advances in nucleotide-based systems, the development of protein-based systems has lagged behind, in spite of the use of protein component expects high catalytic activity and the fine-tuning by amino acid substitution. Artificial PUF proteins fused with yeast poly(A)-binding protein (PAB) activated the translation of targeted *cyclin B1* mRNA in U20S cells, which increased protein abundance by fourfold, while cyclin B1-positive cell numbers were also increased 5-fold^24^. However, the use of the PUF protein is highly restricted in applications with endogenous mRNA because the length of the targeting RNA sequence is 8 or 9 nt, and the binding sequence alteration is not fully programmable.

The results of this study, which showed a high translational enhancement efficiency (sevenfold) and no restriction in the selection of both the length and position of RNA targeting, indicated better or comparable performance of the PPR-based system compared to other translational enhancement techniques; however, the advantages and disadvantages of our system require further clarification in other applications and organisms. To improve and expand the PPR-based technique, it could be useful to fuse the designer PPR protein with YTHDF1 or IF3 as well as to fine-tune the PPR-eIF4G fusion gene by amino acid substitution. Specifically, future therapeutic applications would benefit from the miniaturization of the PPR-based technique.

A remarkable finding of this study was the targeting position-dependent translational enhancement by the PPR-eIF4G fusion protein. This suggests the existence of an unknown alternative translational initiation mechanism. Canonical eukaryotic translational initiation occurs by the recognition of the m^7^G-cap structure of the 5′ end of mRNA by eIF4E and the assembly of eIF4G and eIF4A to form the eIF4F complex by recruiting the 43S ribosome, which scans mRNA from 5′ to 3′ directions to find the translational start codon^39,40^. In this study, we showed that the translational enhancement was found in very limited target positions and that only eIF4G, but not eIF4A and 4E, was functional. In addition, no characteristic features in the sequence context, secondary structure, or the distance from the 5′ end or start codon in the target sequences have been associated with the translational enhancement (Figs. 1E, 5C). It is known that *c-Myc* mRNA is also translated in a cap-independent manner via the IRES^41^. However, the position of the IRES (positions 810–1,078) did not coincide with the translational activation positions in this study (positions 243–782). To gain mechanistic insights into the role of eIF4G, we showed that the eIF3 interaction region is indispensable for the translational enhancement, although other eIF4E and MnK1/2 kinase interaction regions are required for competent activity. The contribution of various interaction regions in eIF4G has also been shown when eIF4G is fused with the λN RNA binding domain^43^. However, translational enhancement was observed in λN-eIF4E fusion gene in this system, as opposed to the PPR fusion genes (Fig. 3). These observations suggest that the translational enhancement by PPR-4G may be based on an anomalistic combination of translational factors. It should be mentioned that since translation activity was not measured directly, alternative mechanisms for the observed results are possible. Further analyses will be required, such as the identification of recruited translational factor(s) coprecipitated with the activation-positive PPR-eIF4G fusion protein and/or ribosome footprint analyses, to obtain information in which step the translation is affected: assembly of initiation complex, recruitment of ribosome, recognition of start codon, and/or elongation.

The PPR-based translational enhancement technique, along with other available technique(s), can resolve the discrepancy between RNA and protein accumulation levels, and therefore would complement conventional transcription enhancement techniques that utilize strong promoters or transcriptional factors^42^. Application of the translational enhancement technique is expected in various practical fields, including the production of useful proteins, such as antibodies in cultured animal cells, and gene therapy for muscular dystrophy caused by mutations in large dystrophin genes, which cannot be treated by replacement therapy. Furthermore, it would be interesting to establish a eukaryotic synthetic gene circuit using both transcriptional and translational enhancement techniques in the near future.

## Materials and methods

### Construction of designer PPR protein gene

The designer PPR protein gene vector was constructed using the golden gate assembly method and PPR3.0 scaffold as described by Yagi et al^29^. Briefly, an intermediate plasmid containing two PPR motifs was chemically synthesized (Azenta Life Science, Chelmsford, MA, USA). The respective PPR scaffolds comprised 144 plasmids and were divided into nine groups (Tw-1 to Tw-9). The respective Tw groups contained 16 plasmids for all the 16 dinucleotide combinations recognized by the two PPR motifs. The designer PPR gene with 18 PPR motifs was assembled using the Tw-1 to Tw-9 plasmids and the expression plasmid of modified pFUS_B1 (#31018; Addgene) via a golden-gate reaction with *Bpi*I and T4-DNA ligase. We mixed 20 ng of each Tw plasmid in 10 μL golden-gate reaction mixture, including 1 μL 10 × ligase buffer (New England Biolabs, Ipswich, MA, USA), 0.5 μL *Bpi*I (Thermo Fisher Scientific, Waltham, MA, USA), 0.5 μL Quick ligase (New England Biolabs), and 25 ng of pFUS_B1 plasmid. The reaction was conducted in a thermal cycler, according to the following temperature cycles: 37 °C for 5 min and 16 °C for 7 min, for 15 cycles. After the reaction, 0.4 μL *Bpi*I was added to the tube and further incubated at 37 °C for 30 min and 75 °C for 6 min. To degrade non-ligated DNA fragments, 0.4 μL of 1 mM ATP and 0.15 μL plasmid-safe nuclease (Epicentre-Lucigen, Middleton, WI, USA) were mixed and incubated at 37 °C for 15 min. Then, 1 μL of the reactions were transformed into XL1-blue-competent cells, planted in LB plate with 50 µg/mL spectinomycin. We used 5 mL of LB medium supplemented with 50 ug/mL spectinomycin as a starting overnight culture grown at 37 °C with agitation, and extracted the plasmids using a Gen Elute HP Plasmid Miniprep Kit (Sigma-Aldrich, St. Louis, MO, USA). The size and sequence of the inserted PPR repeats in the pFUS_B1 plasmid were confirmed by restriction enzyme *Xho*I cleavage and Sanger sequencing using pCR8_Fw (5′-ttgatgcctggcagttccct-3′) and pCR8_Rv (5′-cgaaccgaacaggcttatgt-3′) as primers. The sequences of the PPR proteins used in this study and their RNA target sequences are shown in Supplementary Table 1. The amino acid sequences of all the fusion proteins are shown in Fig. S5.

### Construction of PPR-eIF4G fusion gene containing mutated or truncated eIF4G

The truncated a3c-eIF4G1 fragments carrying the *Esp3*I enzyme site were PCR amplified using the original vector containing the eIF4G DNA fragment pFUSpp_a3c-eIF4G_ori(607–1600) and a KOD FX Neo Kit (Toyobo, Kyoto, Japan) with oligonucleotide primers as follows: forward, 5′-aagcgtctcagtagcggatctggcggagatcccactagactacaaggcat-3′, and reverse, 5′-agacgtctcgtagcgtccgttgtggtcagactcctcctctgcttcacgga-3′ for a3c-eIF4G_t6(653–1600); forward, 5′-aagcgtctcagtagcggatctggcggagatcccactagactacaaggcat-3′, and reverse, 5′-agacgtctcgtagcgtccgttgagtgccctctggccaggggcttccgact-3′ for a3c-eIF4G_t5(653–1438); forward, 5′-aagcgtctcagtagcggatctggcggagatcccactagactacaaggcat-3′, and reverse, 5′-agacgtctcgtagcgtccgttgagtgccctctggccaggggcttccgact-3′ for a3c-eIF4G_t4(653–1131); forward, 5′-aagcgtctcagtagcggatctggcggagaggagaaaaaacgttacgaccg-3′, and reverse, 5′-agacgtctcgtagcgtccgttgagtgccctctggccaggggcttccgact-3′ for eIF4G_t3(607–1438); forward, 5′-aagcgtctcagtagcggatctggcggagaggagaaaaaacgttacgaccg-3′, and reverse, 5′-agacgtctcgtagcgtccgttgagtgccctctggccaggggcttccgact-3′ for eIF4G_t2(607–1131). The amplified DNA fragment was inserted into an empty pFUSpp_a3c vector digested with the *Esp3*I enzyme. The construct of eIF4G_t1(Δ816–953) was made using a PrimeSTAR Mutagenesis Basal Kit (Takara, Kyoto, Japan) with oligonucleotide primers as follows: forward, 5′-ggcctatcgaatggatcagtatttc-3′, and reverse, 5′-tccattcgataggccacagagaagtt- 3′. Amino acid mutated version of PPR-eIF4G was prepared using a PrimeSTAR Mutagenesis Basal Kit (Takara, Kyoto, Japan) with oligonucelotide primers as follows: forward, 5′-gtgcgcgagatcctgaataaactgaca-3′, and reverse, 5′-caggatctcgcgcaccctgcggaatag-3′ for eIF4G_m4(S767E); forward, 5′-gtgcgcgacatcctgaataaactgaca-3′, and reverse, 5′-caggatgtcgcgcaccctgcggaatag-3′ for eIF4G_m3(S767D); forward, 5′-aaactgatcccccagatgttccagcag-3′, and reverse, 5′-ctgggggatcagtttattcaggatgga-3′ for eIF4G_m2(T773I); forward, 5′-ctgcagcgcgtgctggatctgcgaggg-3′, and reverse, 5′-cagcacgcgctgcagcataaagcggat-3′for eIF4G_m1(D982R). The DNA sequences of mutated or truncated eIF4G were verified using an automated sequencer (GENEWIZ; Azenta Life Science, Kyoto, Japan).

### Construction of the PPR-eIF4G fusion gene vector

Prior to the golden-gate reaction assembly, the N-terminus 3 × FLAG tag, PPR, and C-terminus eIF4G genes were integrated into the expression vector of the modified pRL-CMV (#E2261; Addgene) with the *Esp3*I site. We used truncated or mutant version of eIF4G according to the previous study^30,43^ (Fig. S5A). Modified pFUS_B1 vectors (pFUSpp_a3a, pFUSpp_a3b, and pFUSpp_a3c) were used for the 3 × FLAG tag, PPR, and eIF4G in *E. coli*, respectively. The PPR-eIF4G gene was assembled using the pFUSpp_a3a, pFUSpp_a3b, and pFUSpp_a3c plasmids and a modified pRL-CMV plasmid via a golden-gate reaction with *Esp3*I and T4-DNA ligase. We mixed 20 ng each of the pFUSpp_a3a, pFUSpp_a3b, and pFUSpp_a3c plasmids in 1.9 μL golden-gate reaction mixture, including 0.2 μL 10 × ligase buffer, 0.1 μL *Esp3*I, 0.1 μL Quick ligase, and 15 ng pRL-CMV plasmid. The reaction was conducted in a thermal cycler, according to the following temperature cycles: 37 °C for 5 min and 16 °C for 10 min, for 5 cycles. After the reaction, 0.1 μL *Esp3*I, 0.25 μL 10 mM DTT, and 0.25 μL 10 × Tango buffer were added to each tube and further incubated at 37 °C for 60 min and 80 °C for 5 min. Then, 0.5 μL of the reactions were transformed into XL1-blue-competent cells, plated in LB plates with 50 µg/mL ampicillin. We used 5 mL LB medium supplemented with 50 µg/mL spectinomycin as a starting overnight culture grown at 37 °C with agitation, and extracted the plasmids using the Gen Elute HP Plasmid Miniprep Kit. The inserted DNA sequence was confirmed by Sanger sequencing using the primers pRL_Fw (5′-ggtcttactgacatccactttgcc-3′) and pRL_Rv (5′-tcttatcatgtctgctcgaagcg-3′).

The PPR gene fused with eIF4A, eIF4E, and an HA tag was constructed using the same procedure. The sequences are shown in Fig. S5B,C,D.

### Cell culture, transfections, and cell counting

HEK293 cells (ECACC 85120602; KAC, Kyoto, Japan) or Hela cells (ECACC 93021013; KAC) were cultured using Dulbecco’s modified Eagle’s medium (DMEM; FUJIFILM Wako, Osaka, Japan), 10% fetal bovine serum (FBS; Gibco, Thermo Fisher Scientific), and penicillin–streptomycin (Gibco, Thermo Fisher Scientific) at 37 °C with 5% CO_2_. HEK293 or HeLa cells were seeded into 12-well plates at 100,000 cells/well on the day before transfection. The expression plasmid was transfected using FuGENE HD (Promega, Madison, WI, USA), and the cells were incubated for 48 h at 37 °C with 5% CO_2_. For the cell number analysis, cells were collected after 2 d of transfection and counted using a Countess® II FL (Thermo Fisher Scientific).

### RNA Extraction and RT-PCR

Total RNA was extracted using a NucleoSpin RNA clean-up kit (Takara Bio, Shiga, Japan) according to the manufacturer’s instructions. cDNA was generated in a 20 μL reaction mixture containing 10 μL total RNA (500 ng), 6 μL pure water, and 4 μL Mixima cDNA H Minus Synthesis Master Mix (5 × ; Thermo Fisher Scientific). The mixture was incubated at 25 °C for 10 min and 50 °C for 25 min, then heated at 85 °C for 5 min to inactivate the reverse transcriptase. The cDNA was stored at –80 °C until use. PCR was performed using the cDNA as a template with PrimeStar Max DNA polymerase (Takara Bio), using the following primers: p53_Fw (5′-atggaggagccgcagtcagat-3′) and p53_Rv (5′-tcagtctgagtcaggcccttc-3′) for p53 mRNA analysis; c-Myc_Fw (5′-atgcccctcaacgttagcttca-3′) and c-Myc_Rv (5′-aagtttgtgtttcaactgttc-3′) for c-Myc mRNA analysis, and β–actin_Fw (5′-tcgtcgtcgacaacggct-3′) and β–actin_Rv (5′-ggcatgggggagggcata-3′) for β-actin (endogenous control) mRNA analysis.

### Western blot analysis

Total cellular protein was extracted using RIPA lysis buffer and a protease inhibitor cocktail (Sigma-Aldrich). After centrifugation at 18,000×*g* for 10 min, the supernatant was collected and protein concentration was measured using a bicinchoninic acid assay (BCA) kit (Thermo Fisher Scientific). Equal amounts of proteins were subjected to SDS-PAGE and transferred onto PVDF membranes using a discontinuous (Tris/CAPS) buffer system (Bio-Rad Laboratories, Hercules, CA, USA). Western blot analyses were performed using the following antibodies: mouse anti p53 (aa20-25; #MCA1701, Bio-Rad), Anti-p53 antibody [PAb 240] (#ab26, Abcam, Cambridge, UK), Anti-c-Myc antibody [Y69]-ChIP Grade (#ab32072, Abcam), Beta Actin antibody (#CL594-60,008, proteintech, Tokyo, Japan), monoclonal anti-FLAG® M2 antibody (#F3165-1MG, Sigma-Aldrich), and anti-mouse IgG HRP-linked whole antibody sheep (#NA931-1ML, Streptavidin Sepharose High Performance; Cytiva, Tokyo, Japan), Goat Anti-Mouse IgG H&L (HRP) (#ab6789, Abcam), Goat Anti-Rabbit IgG H&L (HRP) (#ab6721, Abcam) according to the manufacturers’ protocols. Chemiluminescent signals were visualized using a ChemiDoc™ Touch instrument (Bio-Rad).

### Homologous time-resolved fluorescence (HTRF) assay for c-Myc protein analysis

c-Myc protein levels were detected by HTRF assay using a human c-Myc cell-based assay kit (Cisbio, Bedford, MA, USA), according to the manufacturer’s protocols.

### ELISA for p53 protein

Hela cells were harvested after 48 h of transfection with the PPR-eIF4G fusion gene (or empty) vector for protein isolation and quantification of p53 protein levels using human p53 ELISA kits (#ab171571, Abcam, Cambridge, UK). The cells were washed once with 1 × PBS and immediately trypsinized in the culture medium. Thereafter, the cells were pelleted by centrifugation at 800 × *g* for 5 min at 4 °C and washed twice with 1 × PBS. The cell pellet was resuspended in 50 μL 1 × Cell Extraction Buffer PTR (#ab171571, Abcam). The protein concentration for each sample was determined using the BCA kit, and the total protein concentration was adjusted by l.5–3 μg/mL for each reaction. The ELISA was performed according to the manufacturer’s instructions. A colorimetric reaction became apparent after the addition of TMB substrate to the protein lysate mixed with protein-specific antibodies in a “sandwich” format in a 96-well plate. We measured p53 protein accumulation at an optical density of 450 nm using a spectrophotometer. A standard curve for p53 protein quantification was constructed using a reference protein from the assay kit.

### Apoptosis assay

The apoptosis assay was performed using a FAM-FLICA® Caspase Assay Kit (Bio-Rad). After 48 h of PPR-eIF4G-fusion-gene transfection, HeLa cells were treated with the fluorescent probe FAM-DEVD-FMK and analyzed under a microscope (DMi8, Leica, Wetzlar, Germany). Quantitative analysis of apoptotic activity was performed by separating fluorescence-positive cells using an EC800 cell analyzer (Sony, Tokyo, Japan).

### RNA–protein complex immunoprecipitation (RIP)

The PPR-eIF4G fusion gene (or empty) vector was transfected into cells via FuGENE HD reagent in 6-well plates. Approximately 800,000 cells were harvested using 0.05% trypsin–EDTA (FUJIFILM Wako) and washed once with 1 × PBS. Total cellular extracts were prepared by lysing the cells in Pierce™ IP Lysis Buffer (Thermo Fisher Scientific), protease inhibitor cocktail, and RNase Inhibitor (Takara Bio) for 20 min on ice. After centrifugation of the lysate at 18,000×*g* and 4 °C for 20 min, the supernatant was extracted for use in the following study. We used 10% of the supernatant as input. Anti-FLAG M2 magnetic beads (#M8823, Sigma-Aldrich) were mixed in the supernatant and incubated for 2 h at 4 °C on a wheel. After washing (three times with IP lysis buffer and once with 1 × PBS buffer), the PPR fusion protein and interacting RNA were eluted by incubation with an excess amount of FLAG peptide (Merck, Darmstadt, Germany) at 4 °C for 30 min. The eluted sample was divided and used for western blot analysis (to detect the PPR-fusion proteins) or RT-PCR (to detect *p53*, *c-Myc*, and *GAPDH* mRNA levels) using the following primers: p53_Fw2 (5′-ctaggatctgactgcggctc-3′), p53_Rv2 (5′-cagaatgcaagaagcccagac-3′); c-Myc_Fw2 (5′-tcctgttggtgaagctaacg-3′), c-Myc_Rv (5′-aggaggccagcttctctgagac-3′); GAPDH_Fw (5′-ctgccgtctagaaaaacc-3′), and GAPDH_Rv (5′-ccaccttcgttgtcatacc-3′).

### Supplementary Information

Supplementary Figures.

Supplementary Table S1.

## Data Availability

All data generated or analyzed during this study are included in this published article and its supplementary information files.

## References

[1] Gaj T, Gersbach CA, Barbas CF (2013). ZFN, TALEN, and CRISPR/Cas-based methods for genome engineering. Trends Biotech..

[2] Chandrasegaran S, Carroll D (2016). Origins of programmable nucleases for genome engineering. J. Mol. Biol..

[3] Wu X (2014). Genome-wide binding of the CRISPR endonuclease Cas9 in mammalian cells. Nat. Biotech..

[4] Hsu PD, Lander ES, Zhang F (2014). Development and applications of CRISPR-Cas9 for genome engineering. Cell.

[5] Komor AC, Badran AH, Liu DR (2017). CRISPR-Based technologies for the manipulation of eukaryotic genomes. Cell.

[6] Quinn JJ, Chang HY (2016). Unique features of long non-coding RNA biogenesis and function. Nat. Rev. Genet..

[7] Pan Q, Shai O, Lee LJ, Frey BJ, Blencowe BJ (2008). Deep surveying of alternative splicing complexity in the human transcriptome by high-throughput sequencing. Nat. Genet..

[8] Zhao H, Wolt JD (2017). Risk associated with off-target plant genome editing and methods for its limitation. Emerg. Topics Life Sci..

[9] Dorsett Y, Tuschl T (2004). siRNAs: Applications in functional genomics and potential as therapeutics. Nat. Rev. Drug Discov..

[10] Bennett CF (2018). Therapeutic antisense oligonucleotides are coming of age. Annu. Rev. Med. 2019.

[11] Crooke ST (2017). Molecular mechanisms of antisense oligonucleotides. Nucl. Acid Ther..

[12] Wilson RC, Doudna JA (2013). Molecular mechanisms of RNA interference. Annu. Rev. Biophys..

[13] Abudayyeh OO (2017). RNA targeting with CRISPR-Cas13. Nature.

[14] Cox DBT (2017). RNA editing with CRISPR-Cas13. Science.

[15] Konermann S (2018). Transcriptome engineering with RNA-targeting type VI-D CRISPR effectors. Cell.

[16] Rauch S, He C, Dickinson BC (2018). Targeted m^6^A reader proteins to study epitranscriptomic regulation of single RNAs. J. Am. Chem. Soc..

[17] Chen Y, Varani G (2013). Engineering RNA-binding proteins for biology. FEBS J..

[18] Johansson HE, Liljas L, Uhlenbeck OC (1997). RNA recognition by the MS2 phage coat protein. Semin. Virol..

[19] Nicholson P, Josi C, Kurosawa H, Yamashita A, Mühlemann O (2014). A novel phosphorylation-independent interaction between SMG6 and UPF1 is essential for human NMD. Nucleic Acids Res..

[20] Wang X, Mclachlan J, Zamore PD, Tanaka Hall TM (2002). Modular recognition of RNA by a human pumilio-homology domain. Cell.

[21] Filipovska A, Razif MFM, NygÅrd KKA, Rackham O (2011). A universal code for RNA recognition by PUF proteins. Nat. Chem. Biol..

[22] Quenault T, Lithgow T, Traven A (2011). PUF proteins: Repression, activation and mRNA localization. Trends Cell Biol..

[23] Choudhury R, Tsai YS, Dominguez D, Wang Y, Wang Z (2012). Engineering RNA endonucleases with customized sequence specificities. Nat. Commun..

[24] Campbell ZT, Valley CT, Wickens M (2014). A protein-RNA specificity code enables targeted activation of an endogenous human transcript. Nat. Struct. Mol. Biol..

[25] Barkan A, Small I (2014). Pentatricopeptide repeat proteins in plants. Annu. Rev. Plant Biol..

[26] Nakamura T, Yagi Y, Kobayashi K (2012). Mechanistic insight into pentatricopeptide repeat proteins as sequence-specific RNA-binding proteins for organellar RNAs in plants. Plant Cell Physiol..

[27] Barkan A (2012). A Combinatorial amino acid code for RNA recognition by pentatricopeptide repeat proteins. PLoS Genet..

[28] Yagi Y, Hayashi S, Kobayashi K, Hirayama T, Nakamura T (2013). Elucidation of the RNA recognition code for pentatricopeptide repeat proteins involved in organelle RNA editing in plants. PLoS ONE.

[29] Yagi Y (2022). Construction of a versatile, programmable RNA-binding protein using designer PPR proteins and its application for splicing control in mammalian cells. Cells.

[30] Kobayashi T, Yagi Y, Nakamura T (2016). Development of genome engineering tools from plant-specific PPR proteins using animal cultured cells. Methods Mol. Biol..

[31] Kastenhuber ER, Lowe SW (2017). Putting p53 in context. Cell.

[32] Saito A, Suga K, Ono-Nakagawa R, Sanada M, Akagawa K (2016). Time lapse imaging analysis of the effect of ER stress modulators on apoptotic cell assessed by caspase3/7 activation in NG108-15 cells. Data Brief..

[33] Yao Y (2015). RNAe: An effective method for targeted protein translation enhancement by artificial non-coding RNA with SINEB2 repeat. Nucl. Acids Res..

[34] Takahashi H (2018). Identification of functional features of synthetic SINEUPs, antisense lncRNAs that specifically enhance protein translation. PLoS ONE.

[35] Toki N (2020). SINEUP long non-coding RNA acts via PTBP1 and HNRNPK to promote translational initiation assemblies. Nucl. Acids Res..

[36] Cao C (2023). Enhancement of protein translation by CRISPR/dCasRx coupled with SINEB2 repeat of noncoding RNAs. Nucl. Acids Res..

[37] Wang X (2015). N^6^-methyladenosine modulates messenger RNA translation efficiency. Cell.

[38] Otoupal PB, Cress BF, Doudna JA, Schoeniger JS (2022). CRISPR-RNAa: targeted activation of translation using dCas13 fusions to translation initiation factors. Nucl. Acids Res..

[39] Leppek K, Das R, Barna M (2018). Functional 5′ UTR mRNA structures in eukaryotic translation regulation and how to find them. Nat. Rev. Mol. Cell Biol..

[40] Jackson RJ, Hellen CUT, Pestova TV (2010). The mechanism of eukaryotic translation initiation and principles of its regulation. Nat. Rev. Mol. Cell Biol..

[41] Nanbru C (1997). Alternative translation of the proto-oncogene c-myc by an internal ribosome entry site. J. Biol. Chem..

[42] Zhang B (2014). Proteogenomic characterization of human colon and rectal cancer. Nature.

[43] Robert F, Cencic R, Cai R, Schmeing TM, Pelletier J (2020). RNA-tethering assay and eIF4G:eIF4A obligate dimer design uncovers multiple eIF4F functional complexes. Nucl. Acids Res..

