# Supplementary material for: Translational enhancement of target endogenous mRNA in mammalian cells using programmable RNA-binding pentatricopeptide repeat proteins
Source: Sci Rep. 2024 Jan 2;14:251. doi: 10.1038/s41598-023-50776-z (PMC10762265; doi:10.1038/s41598-023-50776-z)
Supplement: Supplementary file 1 — Supplementary Figures. [file 41598_2023_50776_MOESM1_ESM.pdf]

Supplementary Information

Translational enhancement of target endogenous mRNA in mammalian cells using programmable RNA-binding pentatricopeptide repeat proteins

Ning Ping, Sayuri Hara-Kuge, Yusuke Yagi,
Tomohiko Kazama and Takahiro Nakamura

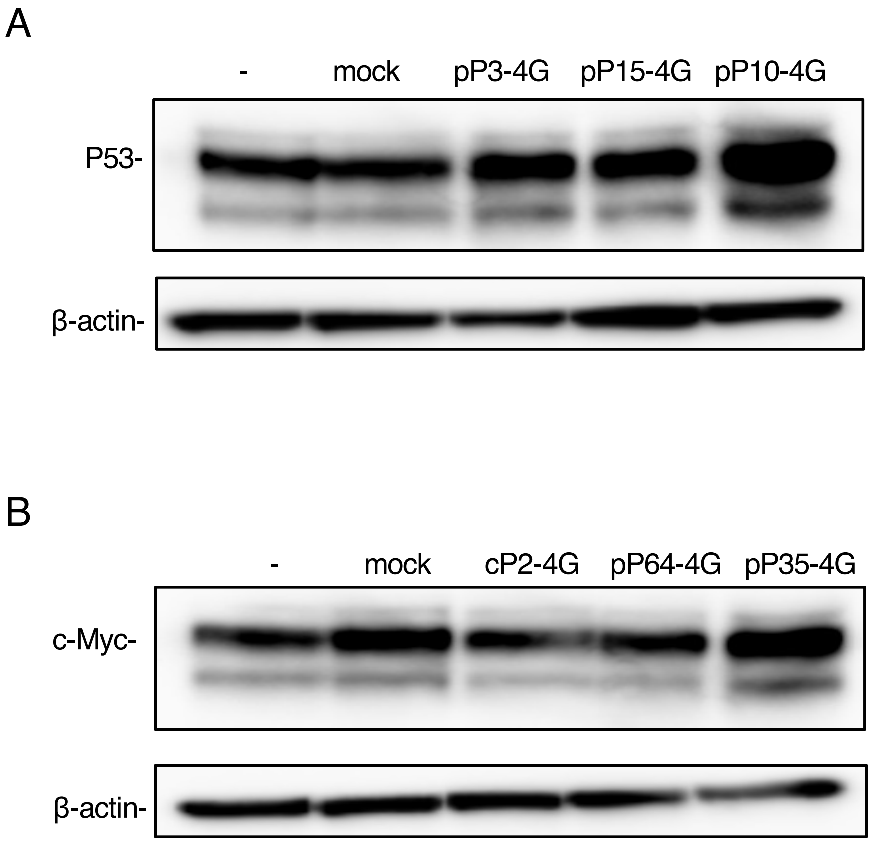

Figure S1. Western blot analysis for p53 or c-Myc protein from Hek293 cell lysates. (A) Western blot analysis to examine p53 protein level without transfection (-), with transfection of empty vector (mock) or PPR-eIF4G fusion gene targeting *p53* mRNA (pP3-4G, pP15-4G, or pP10-4G). β-actin protein was used as an internal control. **(B)** Same as (A), but for PPR-eIF4G fusion gene targeting *c-Myc* mRNA (cP2-4G, cP64-4G and cP35-4G).

A

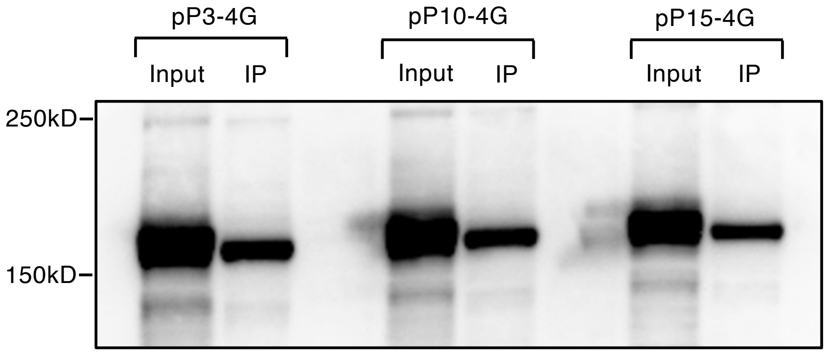

B

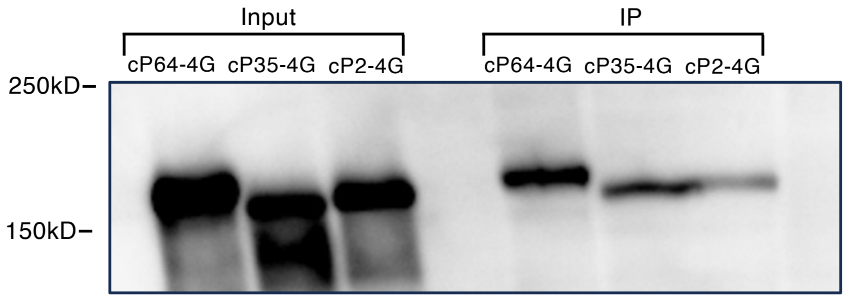

Figure S2. Evaluation of expressed PPR-eIF4G protein for RNA-protein co-immunoprecipitation experiments. (A) The pP3-4G, pP10-4G and pP15-4G proteins in the total lysate (input) or the precipitate (IP) was detected by western blot analysis using anti-FLAG antibody. **(B)** Same as in (A), but for cP64-4G, cP35-4G and cP2-4G fusion proteins.

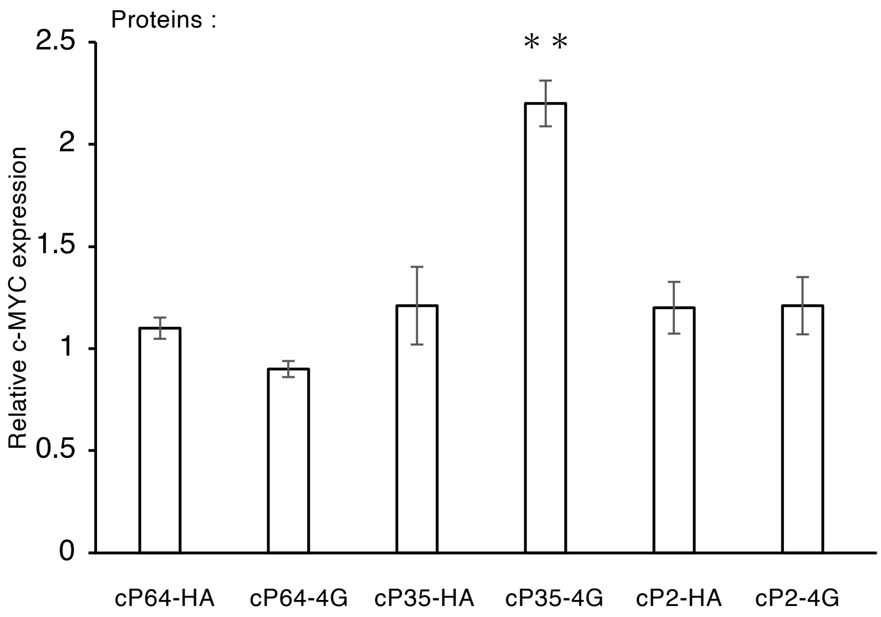

Figure S3. The role of PPR and eIF4G domain in the translational enhancement of *c-Myc* mRNA. The translational enhancement was evaluated with transfection of PPR fusion gene (cP64-HA, cP64-4G, cP35-HA, cP35-4G, cP2-HA or cP2-4G). c-Myc protein level was examined by HTRF assay using Hek293 cells two days after the transfection of PPR fusion gene plasmid. Statistical analysis was conducted using an unpaired, two-tailed Student's t test (N=3; ＊＊: $p<0.01$). Error bar indicates the standard deviation.

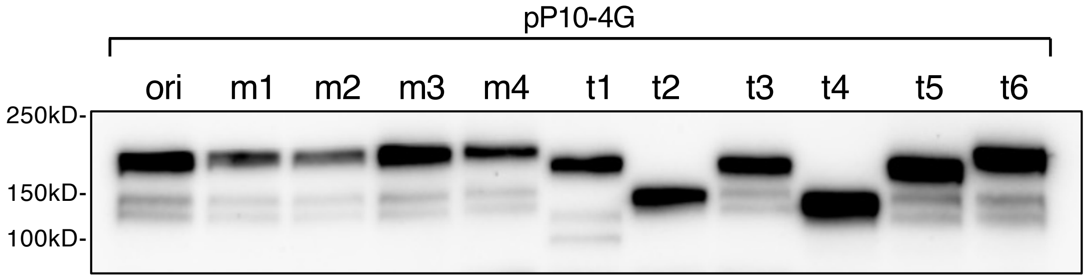

Figure S4. Expressed PPR-eIF4G protein level. The protein level of PPR-eIF4G fusion genes containing mutated or truncated eIF4G (pP10-4G_ori , pP10-4G_m1 to m4, pP10-4G_t1 to t6) was examined by western blot analysis using anti-FLAG antibody.

A

Amino acid sequence of human eIF4G_ori (aa 607 to 1600) protein

EEKKRYDREFLLGFQFIFASMQKPEGLPHISDVVLDKANKTPLRPLDPTRLQGINCGPDFTPSFANLGRTTLSTRGPPRGGPG
GELPRGPQAGLGPRRSQQGPRKEPRKIIATVLMTEDIKLNKAEKAWKPSSKRTAADKDRGEEDADGSKTQDLFRRVRSILNKL
TPQMFQQLMKQVTQLAIDTEERLKGVIDLIFEKAISEPNFSVAYANMCRCLMALKVPTTEKPTVTVNFRKLLLNRCQKEFEKD
KDDDEVFEKKQKEMDEAATAEERGRLKEELEEARDIARRRSLGNIKFIGELFKLKMLTEAIMHDCVVKLLKNHDEESLECLCR
LLTTIGKDLDFEKAKPRMDQYFNQMEKIIKEKKTSSRIRFMLQDVLDLRGSNWVPRRGDQGPKTIDQIHKEAEMEEHREHIKV
QQLMAKGSDKRRGGPPGPPISRGLPLVDDGGWNTVPISKGSRPIDTSRLTKITKPGSIDSNNQLFAPGGRLSWGKGSSGGSGA
KPSDAASEAARPATSTLNRFSALQQAVPTESTDNRRVVQRSSLSRERGEKAGDRGDRLERSERGGDRGDRLDRARTPATKRSF
SKEVEERSRERPSQPEGLRKAASLTEDRDRGRDAVKREAALPPVSPLKAALSEEELEKKSKAIIEEYLHLNDMKEAVQCVQEL
ASPSLLFIFVRHGVESTLERSAIAREHMGQLLHQLLCAGHLSTAQYYQGLYEILELAEDMEIDIPHVWLYLAELVTPILQEGG
VPMGELFREITKPLRPLGKAASLLLEILGLLCKSMGPKKVGTLWREAGLSWKEFLPEGQDIGAFVAEQKVEYTLGEESEAPGQ
RALPSEELNRQLEKLLKEGSSNQRVFDWIEANLSEQQIVSNTLVRALMTAVCYSAIIFETPLRVDVAVLKARAKLLQKYLCDE
QKELQALYALQALVVTLEQPPNLLRMFFDALYDEDVVKEDAFYSWESSKDPAEQQGKGVALKSVTAFFKWLREAEEESDH

Amino acid sequence of human eIF4G_m1 (S767E)

EEKKRYDREFLLGFQFIFASMQKPEGLPHISDVVLDKANKTPLRPLDPTRLQGINCGPDFTPSFANLGRTTLSTRGPPRGGPG
GELPRGPQAGLGPRRSQQGPRKEPRKIIATVLMTEDIKLNKAEKAWKPSSKRTAADKDRGEEDADGSKTQDLFRRVR**E**ILNKL
TPQMFQQLMKQVTQLAIDTEERLKGVIDLIFEKAISEPNFSVAYANMCRCLMALKVPTTEKPTVTVNFRKLLLNRCQKEFEKD
KDDDEVFEKKQKEMDEAATAEERGRLKEELEEARDIARRRSLGNIKFIGELFKLKMLTEAIMHDCVVKLLKNHDEESLECLCR
LLTTIGKDLDFEKAKPRMDQYFNQMEKIIKEKKTSSRIRFMLQDVLDLRGSNWVPRRGDQGPKTIDQIHKEAEMEEHREHIKV
QQLMAKGSDKRRGGPPGPPISRGLPLVDDGGWNTVPISKGSRPIDTSRLTKITKPGSIDSNNQLFAPGGRLSWGKGSSGGSGA
KPSDAASEAARPATSTLNRFSALQQAVPTESTDNRRVVQRSSLSRERGEKAGDRGDRLERSERGGDRGDRLDRARTPATKRSF
SKEVEERSRERPSQPEGLRKAASLTEDRDRGRDAVKREAALPPVSPLKAALSEEELEKKSKAIIEEYLHLNDMKEAVQCVQEL
ASPSLLFIFVRHGVESTLERSAIAREHMGQLLHQLLCAGHLSTAQYYQGLYEILELAEDMEIDIPHVWLYLAELVTPILQEGG
VPMGELFREITKPLRPLGKAASLLLEILGLLCKSMGPKKVGTLWREAGLSWKEFLPEGQDIGAFVAEQKVEYTLGEESEAPGQ
RALPSEELNRQLEKLLKEGSSNQRVFDWIEANLSEQQIVSNTLVRALMTAVCYSAIIFETPLRVDVAVLKARAKLLQKYLCDE
QKELQALYALQALVVTLEQPPNLLRMFFDALYDEDVVKEDAFYSWESSKDPAEQQGKGVALKSVTAFFKWLREAEEESDH

Amino acid sequence of human eIF4G_m2 (S767D)

EEKKRYDREFLLGFQFIFASMQKPEGLPHISDVVLDKANKTPLRPLDPTRLQGINCGPDFTPSFANLGRTTLSTRGPPRGGPG
GELPRGPQAGLGPRRSQQGPRKEPRKIIATVLMTEDIKLNKAEKAWKPSSKRTAADKDRGEEDADGSKTQDLFRRVR**D**ILNKL
TPQMFQQLMKQVTQLAIDTEERLKGVIDLIFEKAISEPNFSVAYANMCRCLMALKVPTTEKPTVTVNFRKLLLNRCQKEFEKD
KDDDEVFEKKQKEMDEAATAEERGRLKEELEEARDIARRRSLGNIKFIGELFKLKMLTEAIMHDCVVKLLKNHDEESLECLCR
LLTTIGKDLDFEKAKPRMDQYFNQMEKIIKEKKTSSRIRFMLQDVLDLRGSNWVPRRGDQGPKTIDQIHKEAEMEEHREHIKV
QQLMAKGSDKRRGGPPGPPISRGLPLVDDGGWNTVPISKGSRPIDTSRLTKITKPGSIDSNNQLFAPGGRLSWGKGSSGGSGA
KPSDAASEAARPATSTLNRFSALQQAVPTESTDNRRVVQRSSLSRERGEKAGDRGDRLERSERGGDRGDRLDRARTPATKRSF
SKEVEERSRERPSQPEGLRKAASLTEDRDRGRDAVKREAALPPVSPLKAALSEEELEKKSKAIIEEYLHLNDMKEAVQCVQEL
ASPSLLFIFVRHGVESTLERSAIAREHMGQLLHQLLCAGHLSTAQYYQGLYEILELAEDMEIDIPHVWLYLAELVTPILQEGG
VPMGELFREITKPLRPLGKAASLLLEILGLLCKSMGPKKVGTLWREAGLSWKEFLPEGQDIGAFVAEQKVEYTLGEESEAPGQ
RALPSEELNRQLEKLLKEGSSNQRVFDWIEANLSEQQIVSNTLVRALMTAVCYSAIIFETPLRVDVAVLKARAKLLQKYLCDE
QKELQALYALQALVVTLEQPPNLLRMFFDALYDEDVVKEDAFYSWESSKDPAEQQGKGVALKSVTAFFKWLREAEEESDH

Amino acid sequence of human eIF4G_m3 (T773I)

EEKKRYDREFLLGFQFIFASMQKPEGLPHISDVVLDKANKTPLRPLDPTRLQGINCGPDFTPSFANLGRTTLSTRGPPRGGPG
GELPRGPQAGLGPRRSQQGPRKEPRKIIATVLMTEDIKLNKAEKAWKPSSKRTAADKDRGEEDADGSKTQDLFRRVRDILNKL
IPQMFQQLMKQVTQLAIDTEERLKGVIDLIFEKAISEPNFSVAYANMCRCLMALKVPTTEKPTVTVNFRKLLLNRCQKEFEKD
KDDDEVFEKKQKEMDEAATAEERGRLKEELEEARDIARRRSLGNIKFIGELFKLKMLTEAIMHDCVVKLLKNHDEESLECLCR
LLTTIGKDLDFEKAKPRMDQYFNQMEKIIKEKKTSSRIRFMLQDVLDLRGSNWVPRRGDQGPKTIDQIHKEAEMEEHREHIKV
QQLMAKGSDKRRGGPPGPPISRGLPLVDDGGWNTVPISKGSRPIDTSRLTKITKPGSIDSNNQLFAPGGRLSWGKGSSGGSGA
KPSDAASEAARPATSTLNRFSALQQAVPTESTDNRRVVQRSSLSRERGEKAGDRGDRLERSERGGDRGDRLDRARTPATKRSF
SKEVEERSRERPSQPEGLRKAASLTEDRDRGRDAVKREAALPPVSPLKAALSEEELEKKSKAIIEEYLHLNDMKEAVQCVQEL
ASPSLLFIFVRHGVESTLERSAIAREHMGQLLHQLLCAGHLSTAQYYQGLYEILELAEDMEIDIPHVWLYLAELVTPILQEGG
VPMGELFREITKPLRPLGKAASLLLEILGLLCKSMGPKKVGTLWREAGLSWKEFLPEGQDIGAFVAEQKVEYTLGEESEAPGQ
RALPSEELNRQLEKLLKEGSSNQRVFDWIEANLSEQQIVSNTLVRALMTAVCYSAIIFETPLRVDVAVLKARAKLLQKYLCDE
QKELQALYALQALVVTLEQPPNLLRMFFDALYDEDVVKEDAFYSWESSKDPAEQQGKGVALKSVTAFFKWLREAEEESDH

Amino acid sequence of human eIF4G_m3 (D982R)

EEKKRYDREFLLGFQFIFASMQKPEGLPHISDVVLDKANKTPLRPLDPTRLQGINCGPDFTPSFANLGRTTLSTRGPPRGGPG
GELPRGPQAGLGPRRSQQGPRKEPRKIIATVLMTEDIKLNKAEKAWKPSSKRTAADKDRGEEDADGSKTQDLFRRVRDILNKL
IPQMFQQLMKQVTQLAIDTEERLKGVIDLIFEKAISEPNFSVAYANMCRCLMALKVPTTEKPTVTVNFRKLLLNRCQKEFEKD
KDDDEVFEKKQKEMDEAATAEERGRLKEELEEARDIARRRSLGNIKFIGELFKLKMLTEAIMHDCVVKLLKNHDEESLECLCR
LLTTIGKDLDFEKAKPRMDQYFNQMEKIIKEKKTSSRIRFMLQ**R**VLDLRGSNWVPRRGDQGPKTIDQIHKEAEMEEHREHIKV
QQLMAKGSDKRRGGPPGPPISRGLPLVDDGGWNTVPISKGSRPIDTSRLTKITKPGSIDSNNQLFAPGGRLSWGKGSSGGSGA
KPSDAASEAARPATSTLNRFSALQQAVPTESTDNRRVVQRSSLSRERGEKAGDRGDRLERSERGGDRGDRLDRARTPATKRSF
SKEVEERSRERPSQPEGLRKAASLTEDRDRGRDAVKREAALPPVSPLKAALSEEELEKKSKAIIEEYLHLNDMKEAVQCVQEL
ASPSLLFIFVRHGVESTLERSAIAREHMGQLLHQLLCAGHLSTAQYYQGLYEILELAEDMEIDIPHVWLYLAELVTPILQEGG
VPMGELFREITKPLRPLGKAASLLLEILGLLCKSMGPKKVGTLWREAGLSWKEFLPEGQDIGAFVAEQKVEYTLGEESEAPGQ
RALPSEELNRQLEKLLKEGSSNQRVFDWIEANLSEQQIVSNTLVRALMTAVCYSAIIFETPLRVDVAVLKARAKLLQKYLCDE
QKELQALYALQALVVTLEQPPNLLRMFFDALYDEDVVKEDAFYSWESSKDPAEQQGKGVALKSVTAFFKWLREAEEESDH

Amino acid sequence of human eIF4G_t1 (aa 653 to 1600) protein
DPTRLQGINCGPDFTPSFANLGRTTLSTRGPPRGGPGGELPRGPQAGLGPRRSQQGPRKEPRKIIATVLMTEDIKLNKAEKAW
KPSSKRTAADKDRGEEDADGSKTQDLFRRVRSILNKLTPQMFQQLMKQVTQLAIDTEERLKGVIDLIFEKAISEPNFSVAYAN
MCRCLMALKVPTTEKPTVTVNFRKLLLNRCQKEFEKDKDDDEVFEKKQKEMDEAATAEERGRLKEELEEARDIARRRSLGNIK
FIGELFKLKMLTEAIMHDCVVKLLKNHDEESLECLCRLLTTIGKDLDFEKAKPRMDQYFNQMEKIIKEKKTSSRIRFMLQDVL
DLRGSNWVPRRGDQGPKTIDQIHKEAEMEEHREHIKVQQLMAKGSDKRRGGPPGPPISRGLPLVDDGGWNTVPISKGSRPIDT
SRLTKITKPGSIDSNNQLFAPGGRLSWGKGSSGGSGAKPSDAASEAARPATSTLNRFSALQQAVPTESTDNRRVVQRSSLSRE
RGEKAGDRGDRLERSERGGDRGDRLDRARTPATKRSFSKEVEERSRERPSQPEGLRKAASLTEDRDRGRDAVKREAALPPVSP
LKAALSEEELEKKSKAIIEEYLHLNDMKEAVQCVQELASPSLLFIFVRHGVESTLERSAIAREHMGQLLHQLLCAGHLSTAQY
YQGLYEILELAEDMEIDIPHVWLYLAELVTPILQEGGVPMGELFREITKPLRPLGKAASLLLEILGLLCKSMGPKKVGTLWRE
AGLSWKEFLPEGQDIGAFVAEQKVEYTLGEESEAPGQRALPSEELNRQLEKLLKEGSSNQRVFDWIEANLSEQQIVSNTLVRA
LMTAVCYSAIIFETPLRVDVAVLKARAKLLQKYLCDEQKELQALYALQALVVTLEQPPNLLRMFFDALYDEDVVKEDAFYSWE
SSKDPAEQQGKGVALKSVTAFFKWLREAEEESDH

Amino acid sequence of human eIF4G_t2 (aa 653 to 1438) protein
DPTRLQGINCGPDFTPSFANLGRTTLSTRGPPRGGPGGELPRGPQAGLGPRRSQQGPRKEPRKIIATVLMTEDIKLNKAEKAW
KPSSKRTAADKDRGEEDADGSKTQDLFRRVRSILNKLTPQMFQQLMKQVTQLAIDTEERLKGVIDLIFEKAISEPNFSVAYAN
MCRCLMALKVPTTEKPTVTVNFRKLLLNRCQKEFEKDKDDDEVFEKKQKEMDEAATAEERGRLKEELEEARDIARRRSLGNIK
FIGELFKLKMLTEAIMHDCVVKLLKNHDEESLECLCRLLTTIGKDLDFEKAKPRMDQYFNQMEKIIKEKKTSSRIRFMLQDVL
DLRGSNWVPRRGDQGPKTIDQIHKEAEMEEHREHIKVQQLMAKGSDKRRGGPPGPPISRGLPLVDDGGWNTVPISKGSRPIDT
SRLTKITKPGSIDSNNQLFAPGGRLSWGKGSSGGSGAKPSDAASEAARPATSTLNRFSALQQAVPTESTDNRRVVQRSSLSRE
RGEKAGDRGDRLERSERGGDRGDRLDRARTPATKRSFSKEVEERSRERPSQPEGLRKAASLTEDRDRGRDAVKREAALPPVSP
LKAALSEEELEKKSKAIIEEYLHLNDMKEAVQCVQELASPSLLFIFVRHGVESTLERSAIAREHMGQLLHQLLCAGHLSTAQY
YQGLYEILELAEDMEIDIPHVWLYLAELVTPILQEGGVPMGELFREITKPLRPLGKAASLLLEILGLLCKSMGPKKVGTLWRE
AGLSWKEFLPEGQDIGAFVAEQKVEYTLGEESEAPGQRALPSEELNRQL

Amino acid sequence of human eIF4G_t3 (aa 653 to 1131) protein
DPTRLQGINCGPDFTPSFANLGRTTLSTRGPPRGGPGGELPRGPQAGLGPRRSQQGPRKEPRKIIATVLMTEDIKLNKAEKAW
KPSSKRTAADKDRGEEDADGSKTQDLFRRVRSILNKLTPQMFQQLMKQVTQLAIDTEERLKGVIDLIFEKAISEPNFSVAYAN
MCRCLMALKVPTTEKPTVTVNFRKLLLNRCQKEFEKDKDDDEVFEKKQKEMDEAATAEERGRLKEELEEARDIARRRSLGNIK
FIGELFKLKMLTEAIMHDCVVKLLKNHDEESLECLCRLLTTIGKDLDFEKAKPRMDQYFNQMEKIIKEKKTSSRIRFMLQDVL
DLRGSNWVPRRGDQGPKTIDQIHKEAEMEEHREHIKVQQLMAKGSDKRRGGPPGPPISRGLPLVDDGGWNTVPISKGSRPIDT
SRLTKITKPGSIDSNNQLFAPGGRLSWGKGSSGGSGAKPSDAASEAARPATSTLNRFSALQQAVPTESTDNRRVVQ

Amino acid sequence of human eIF4G_t4 (aa 607 to 1438) protein
EEKKRYDREFLLGFQFIFASMQKPEGLPHISDVVLDKANKTPLRPLDPTRLQGINCGPDFTPSFANLGRTTLSTRGPPRGGPG
GELPRGPQAGLGPRRSQQGPRKEPRKIIATVLMTEDIKLNKAEKAWKPSSKRTAADKDRGEEDADGSKTQDLFRRVRSILNKL
TPQMFQQLMKQVTQLAIDTEERLKGVIDLIFEKAISEPNFSVAYANMCRCLMALKVPTTEKPTVTVNFRKLLLNRCQKEFEKD
KDDDEVFEKKQKEMDEAATAEERGRLKEELEEARDIARRRSLGNIKFIGELFKLKMLTEAIMHDCVVKLLKNHDEESLECLCR
LLTTIGKDLDFEKAKPRMDQYFNQMEKIIKEKKTSSRIRFMLQDVLDLRGSNWVPRRGDQGPKTIDQIHKEAEMEEHREHIKV
QQLMAKGSDKRRGGPPGPPISRGLPLVDDGGWNTVPISKGSRPIDTSRLTKITKPGSIDSNNQLFAPGGRLSWGKGSSGGSGA
KPSDAASEAARPATSTLNRFSALQQAVPTESTDNRRVVQRSSLSRERGEKAGDRGDRLERSERGGDRGDRLDRARTPATKRSF
SKEVEERSRERPSQPEGLRKAASLTEDRDRGRDAVKREAALPPVSPLKAALSEEELEKKSKAIIEEYLHLNDMKEAVQCVQEL
ASPSLLFIFVRHGVESTLERSAIAREHMGQLLHQLLCAGHLSTAQYYQGLYEILELAEDMEIDIPHVWLYLAELVTPILQEGG
VPMGELFREITKPLRPLGKAASLLLEILGLLCKSMGPKKVGTLWREAGLSWKEFLPEGQDIGAFVAEQKVEYTLGEESEAPGQ
RALPSEELNRQL

Amino acid sequence of human eIF4G_t5 (aa 607 to 1131) protein
EEKKRYDREFLLGFQFIFASMQKPEGLPHISDVVLDKANKTPLRPLDPTRLQGINCGPDFTPSFANLGRTTLSTRGPPRGGPG
GELPRGPQAGLGPRRSQQGPRKEPRKIIATVLMTEDIKLNKAEKAWKPSSKRTAADKDRGEEDADGSKTQDLFRRVRSILNKL
TPQMFQQLMKQVTQLAIDTEERLKGVIDLIFEKAISEPNFSVAYANMCRCLMALKVPTTEKPTVTVNFRKLLLNRCQKEFEKD
KDDDEVFEKKQKEMDEAATAEERGRLKEELEEARDIARRRSLGNIKFIGELFKLKMLTEAIMHDCVVKLLKNHDEESLECLCR
LLTTIGKDLDFEKAKPRMDQYFNQMEKIIKEKKTSSRIRFMLQDVLDLRGSNWVPRRGDQGPKTIDQIHKEAEMEEHREHIKV
QQLMAKGSDKRRGGPPGPPISRGLPLVDDGGWNTVPISKGSRPIDTSRLTKITKPGSIDSNNQLFAPGGRLSWGKGSSGGSGA
KPSDAASEAARPATSTLNRFSALQQAVPTESTDNRRVVQ

Amino acid sequence of human eIF4G_t6 (aa Δ816 to 953 of deletion) protein
EEKKRYDREFLLGFQFIFASMQKPEGLPHISDVVLDKANKTPLRPLDPTRLQGINCGPDFTPSFANLGRTTLSTRGPPRGGPG
GELPRGPQAGLGPRRSQQGPRKEPRKIIATVLMTEDIKLNKAEKAWKPSSKRTAADKDRGEEDADGSKTQDLFRRVRSILNKL
TPQMFQQLMKQVTQLAIDTEERLKGVIDLIFEKAISEPNFSVAYRMDQYFNQMEKIIKEKKTSSRIRFMLQDVLDLRGSNWVP
RRGDQGPKTIDQIHKEAEMEEHREHIKVQQLMAKGSDKRRGGPPGPPISRGLPLVDDGGWNTVPISKGSRPIDTSRLTKITKP
GSIDSNNQLFAPGGRLSWGKGSSGGSGAKPSDAASEAARPATSTLNRFSALQQAVPTESTDNRRVVQRSSLSRERGEKAGDRG
DRLERSERGGDRGDRLDRARTPATKRSFSKEVEERSRERPSQPEGLRKAASLTEDRDRGRDAVKREAALPPVSPLKAALSEEE
LEKKSKAIIEEYLHLNDMKEAVQCVQELASPSLLFIFVRHGVESTLERSAIAREHMGQLLHQLLCAGHLSTAQYYQGLYEILE
LAEDMEIDIPHVWLYLAELVTPILQEGGVPMGELFREITKPLRPLGKAASLLLEILGLLCKSMGPKKVGTLWREAGLSWKEFL
PEGQDIGAFVAEQKVEYTLGEESEAPGQRALPSEELNRQLEKLLKEGSSNQRVFDWIEANLSEQQIVSNTLVRALMTAVCYSA
IIFETPLRVDVAVLKARAKLLQKYLCDEQKELQALYALQALVVTLEQPPNLLRMFFDALYDEDVVKEDAFYSWESSKDPAEQQ
GKGVALKSVTAFFKWLREAEEESDH

B

Amino acid sequence of human eIF4E (aa 2 to 217)

ATVEPETTPTPNPPTTEEEKTESNQEVANPEHYIKHPLQNRWALWFFKNDKSKTWQANLRLISKFDTVEDFWALYNHIQLSSN
LMPGCDYSLFKDGIEPMWEDEKNKRGGRWLITLNKQQRRSDLDRFWLETLLCLIGESFDDYSDDVCGAVVNVRAKGDKIAIWT
TECENREAVTHIGRVYKERLGLPPKIVIGYQSHADTATKSGSTTKNRFV

C

Amino acid sequence of human eIF4A1 (aa 2 to 406)

SASQDSRSRDNGPDGMEPEGVIESNWNEIVDSFDDMNLSESLLRGIYAYGFEKPSAIQQRAILPCIKGYDVIAQAQSGTGKTA
TFAISILQQIELDLKATQALVLAPTRELAQQIQKVVMALGDYMGASCHACIGGTNVRAEVQKLQMEAPHIIVGTPGRVFDMLN
RRYLSPKYIKMFVLDEADEMLSRGFKDQIYDIFQKLNSNTQVVLLSATMPSDVLEVTKKFMRDPIRILVKKEELTLEGIRQFY
INVEREEWKLDTLCDLYETLTITQAVIFINTRRKVDWLTEKMHARDFTVSAMHGDMDQKERDVIMREFRSGSSRVLITTDLLA
RGIDVQQVSLVINYDLPTNRENYIHRIGRGGRFGRKGVAINMVTEEDKRTLRDIETFYNTSIEEMPLNVADL

D

Amino acid sequence of HA tag

YPYDVPDYA

Figure S5. Amino acid sequence of protein domains fused to PPR protein. Amino acid sequence of eIF4G (ori, m1to m4, t1 to t6) , 4E, and 4A, and HA tag proteins are shown in A, B, C, and D, respectively.

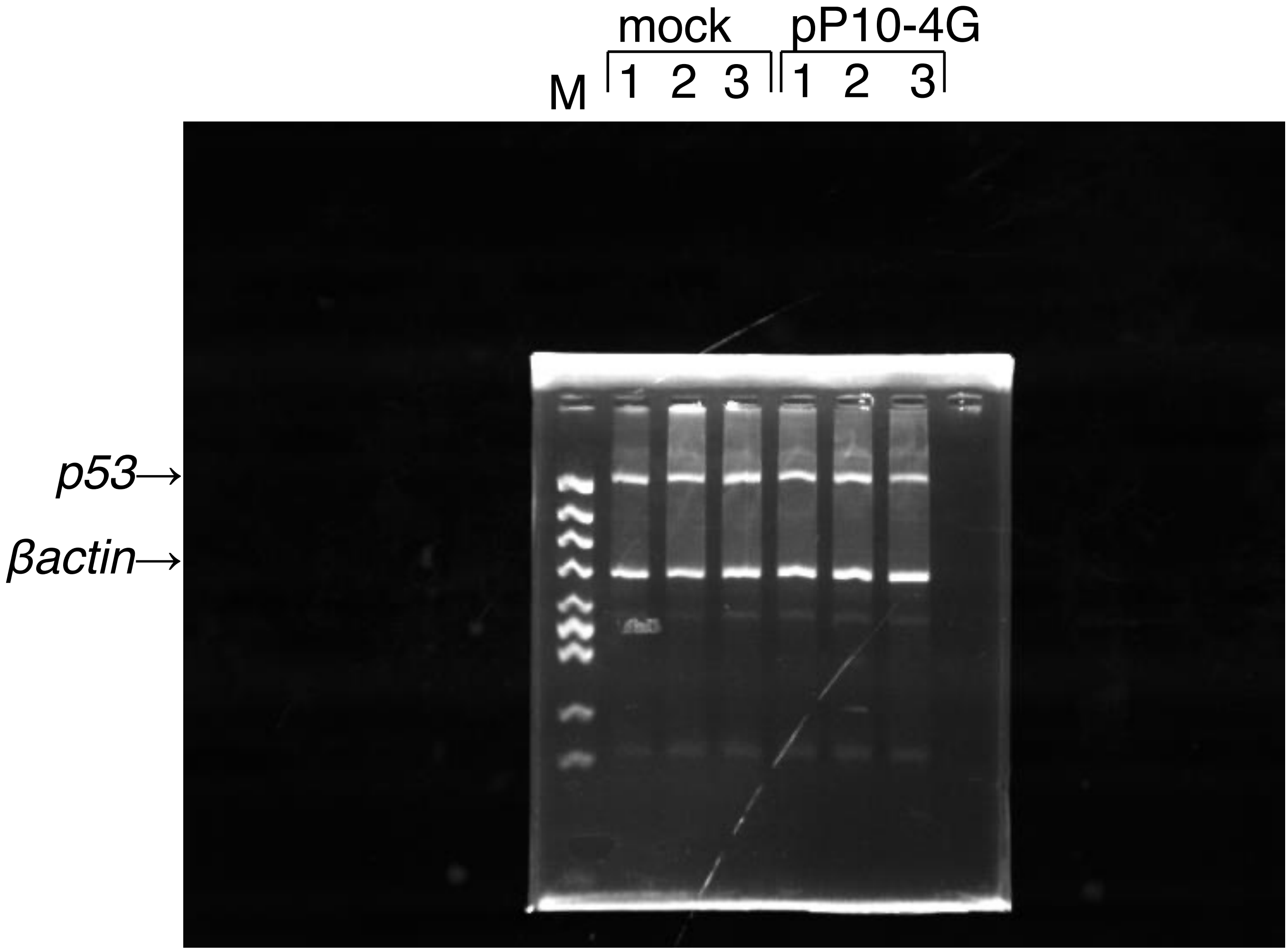

Gel image for Figure 1D

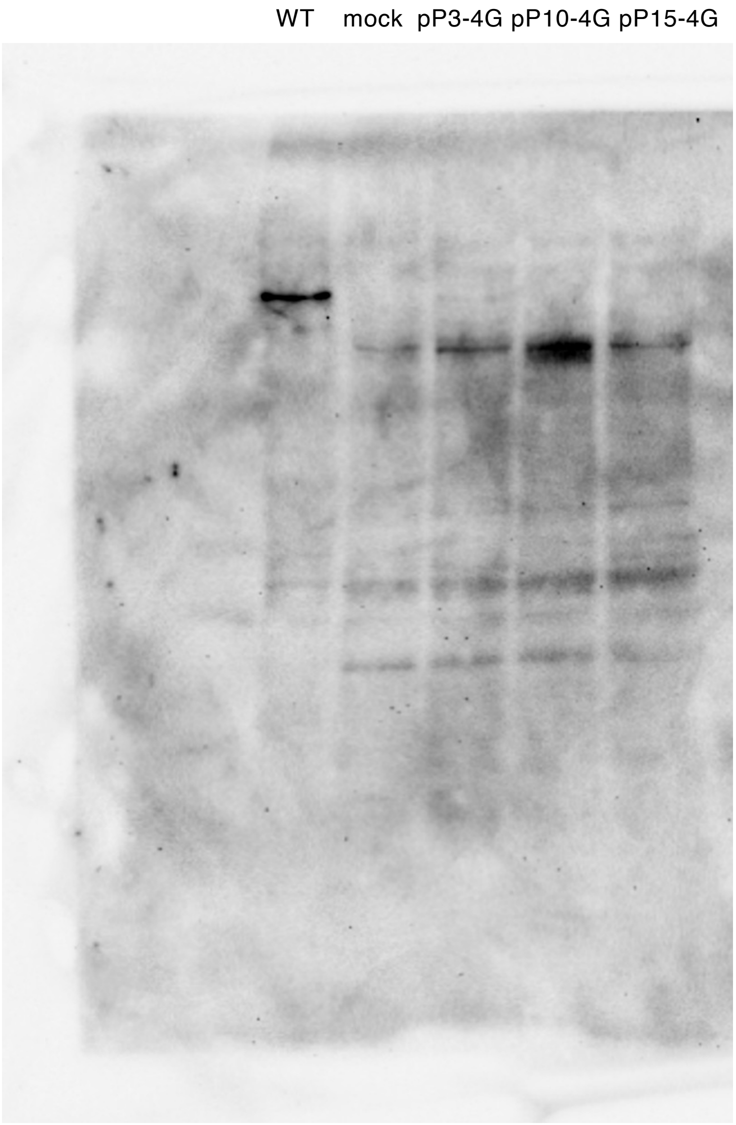

Gel image for Figure 1F (upper panel)

WT mock pP3-4G pP10-4G pP15-4G

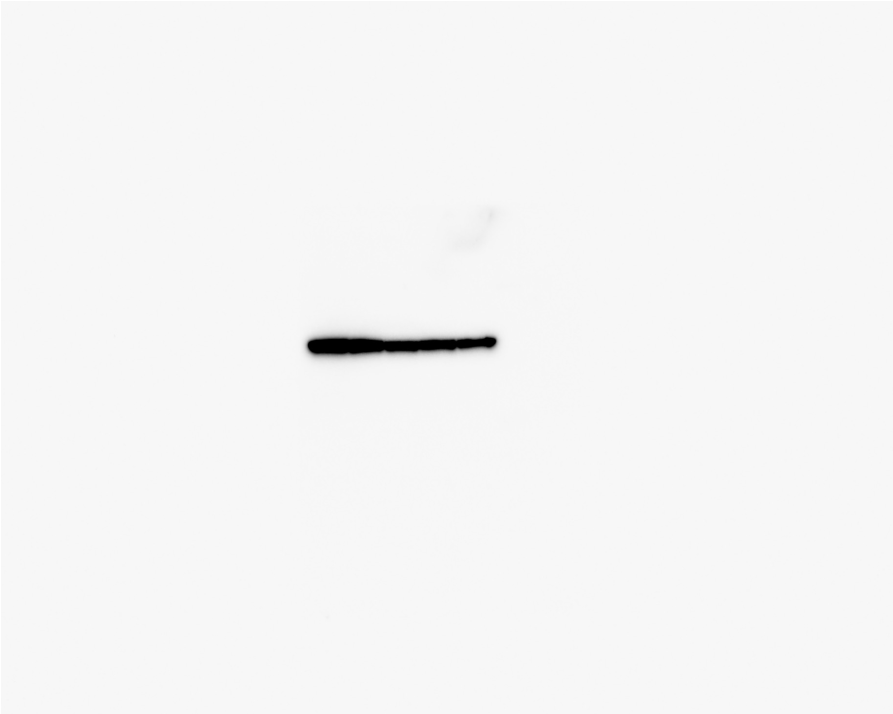

Gel image for Figure 1F (lower panel)

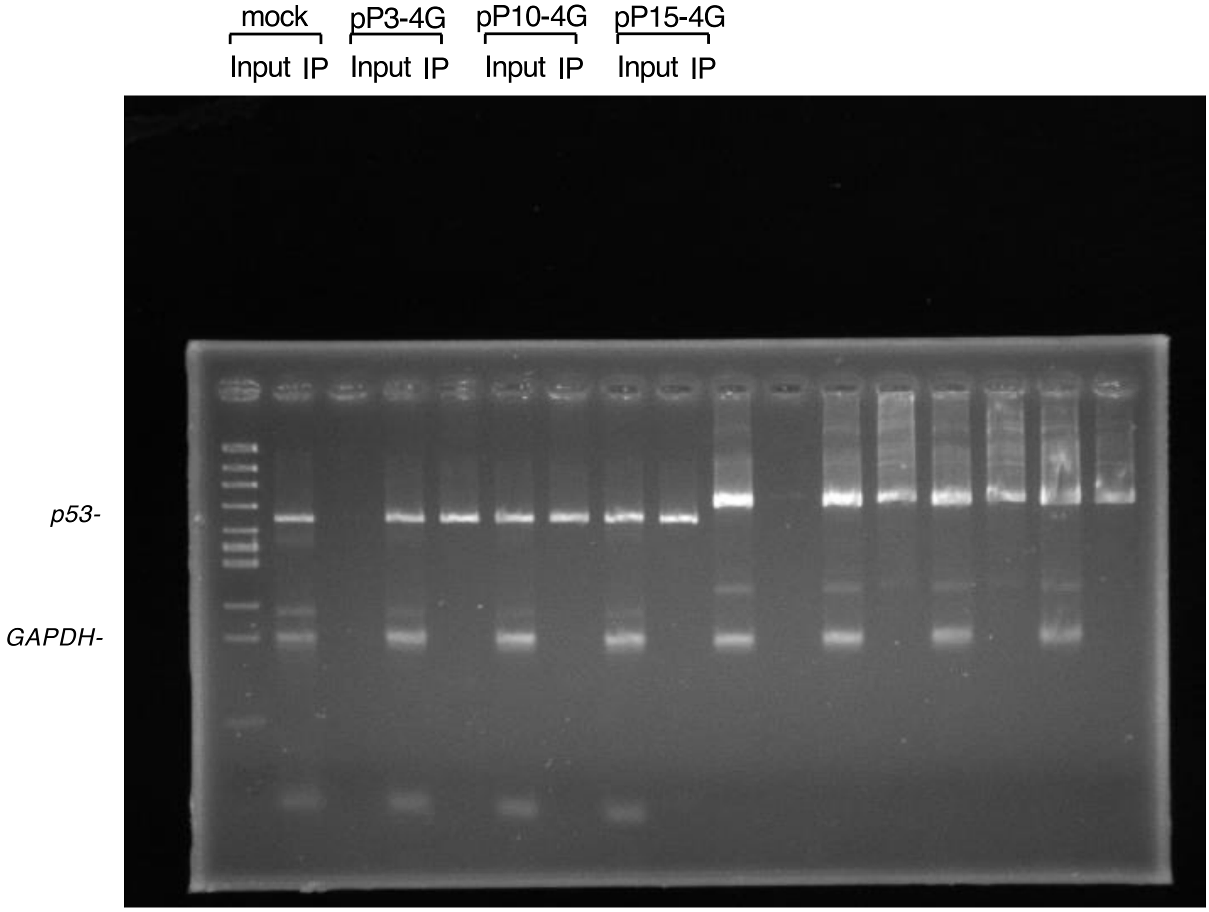

Gel image for Figure 1G

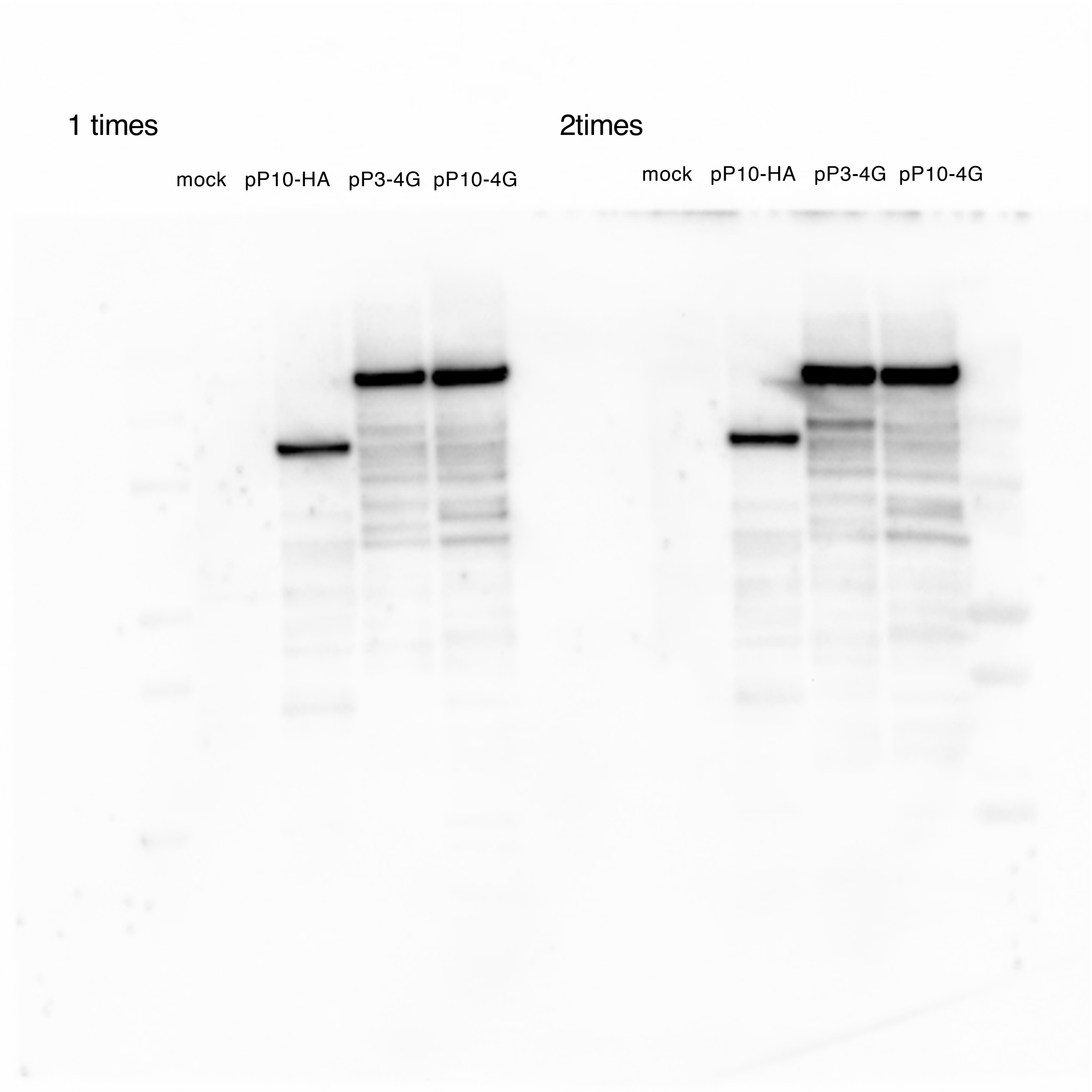

Gel image for Figure 3C

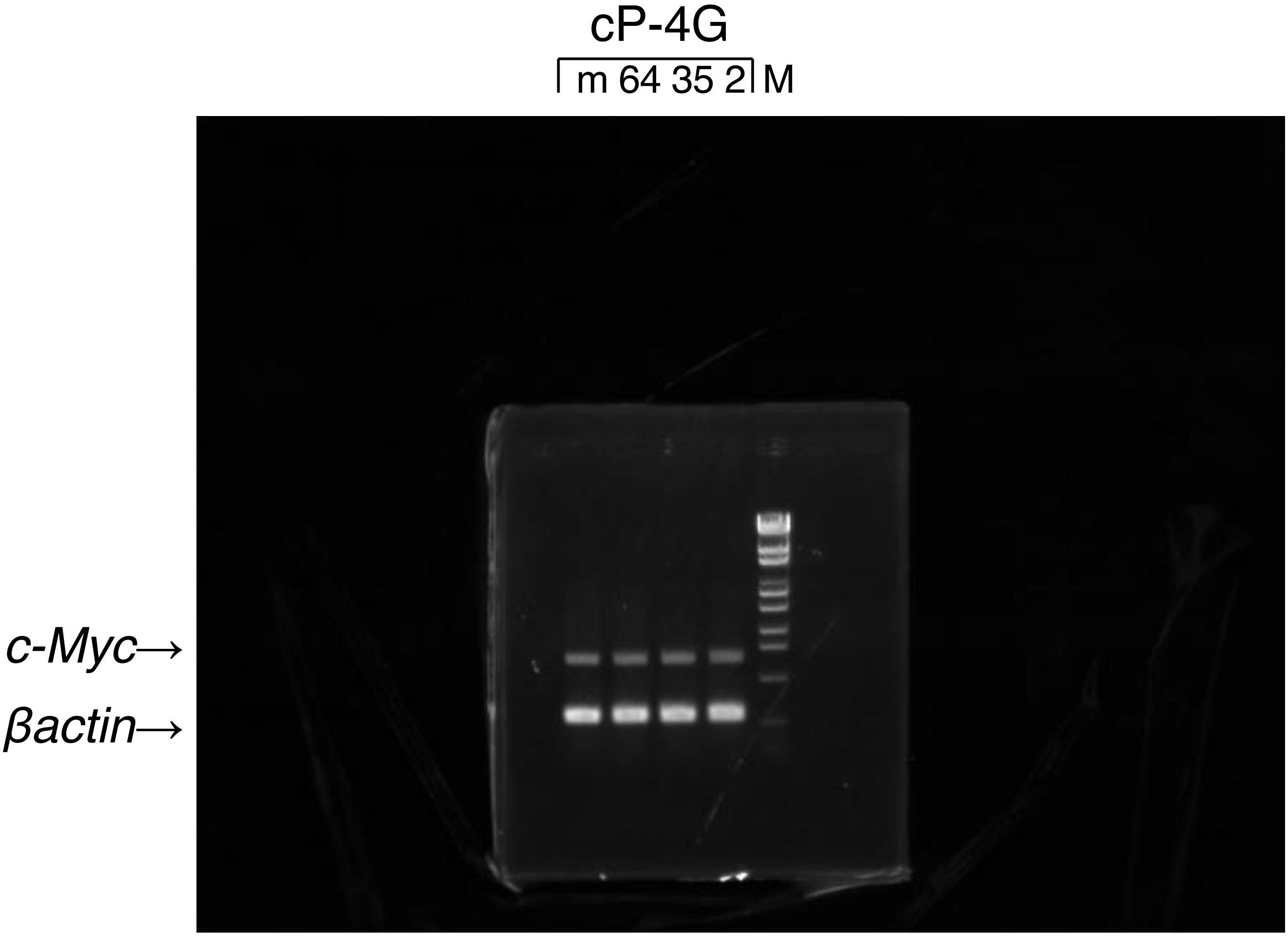

Gel image for Figure 5D

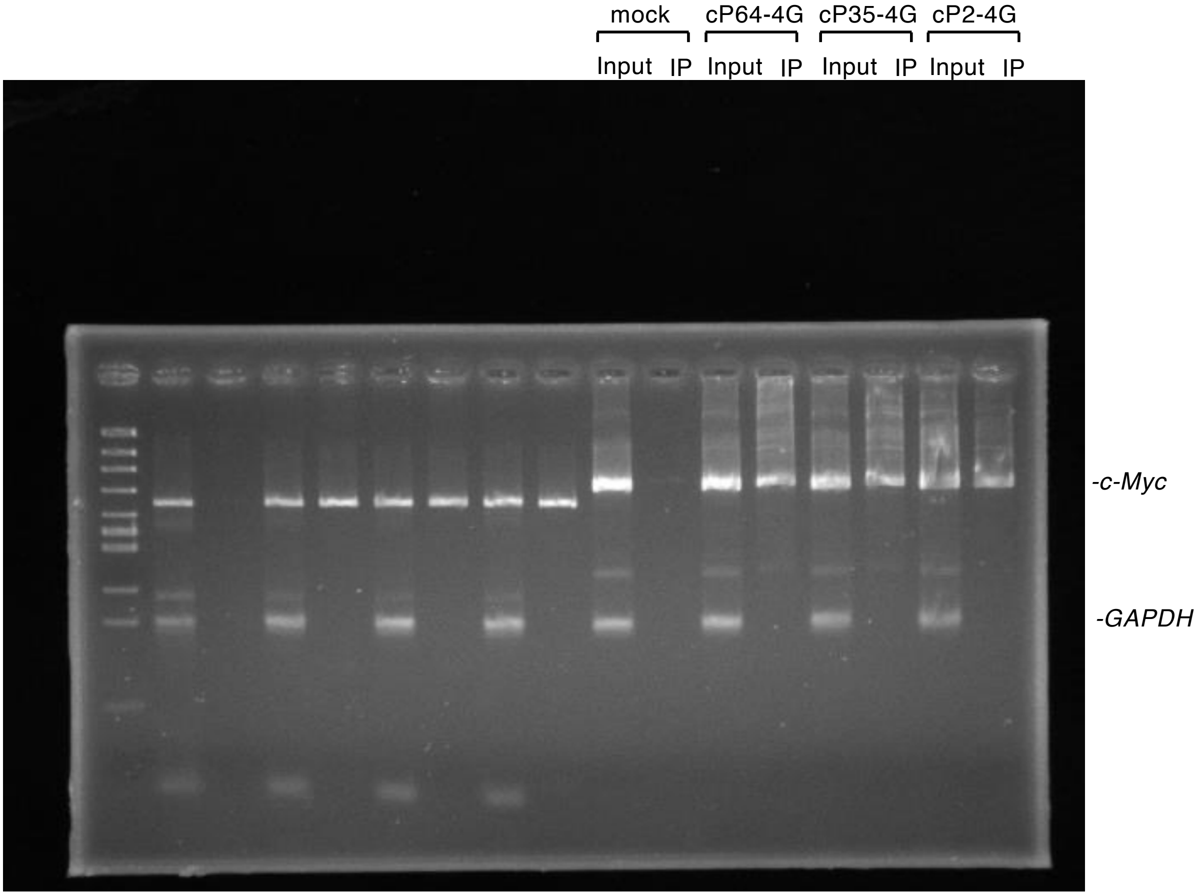

Gel image for Figure 5E

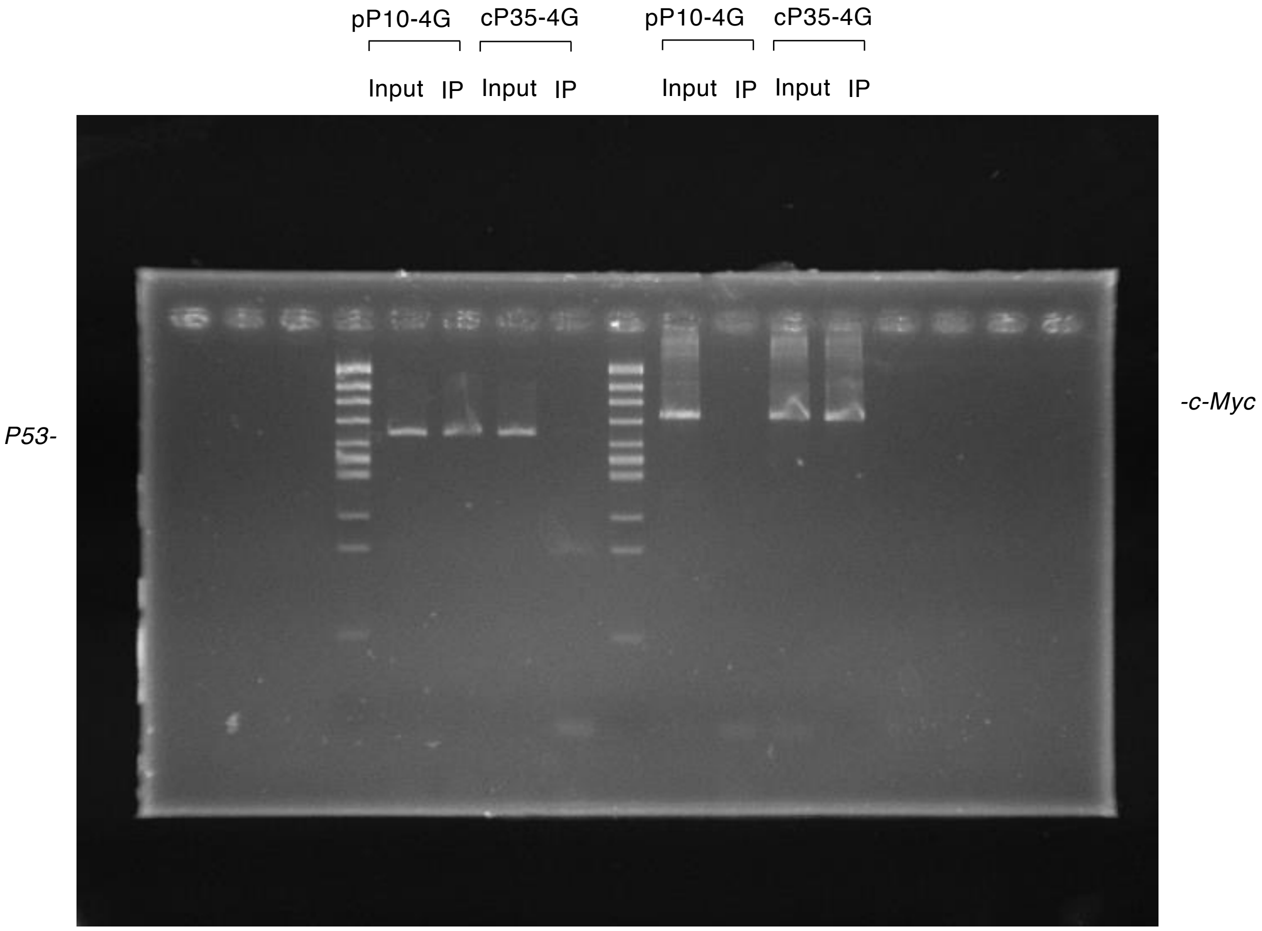

Gel image for Figure 5F

(A)
WT mock pP3-4G pP15-4G pP10-4G

(B)
WT mock cP2-4G pP64-4G pP35-4G

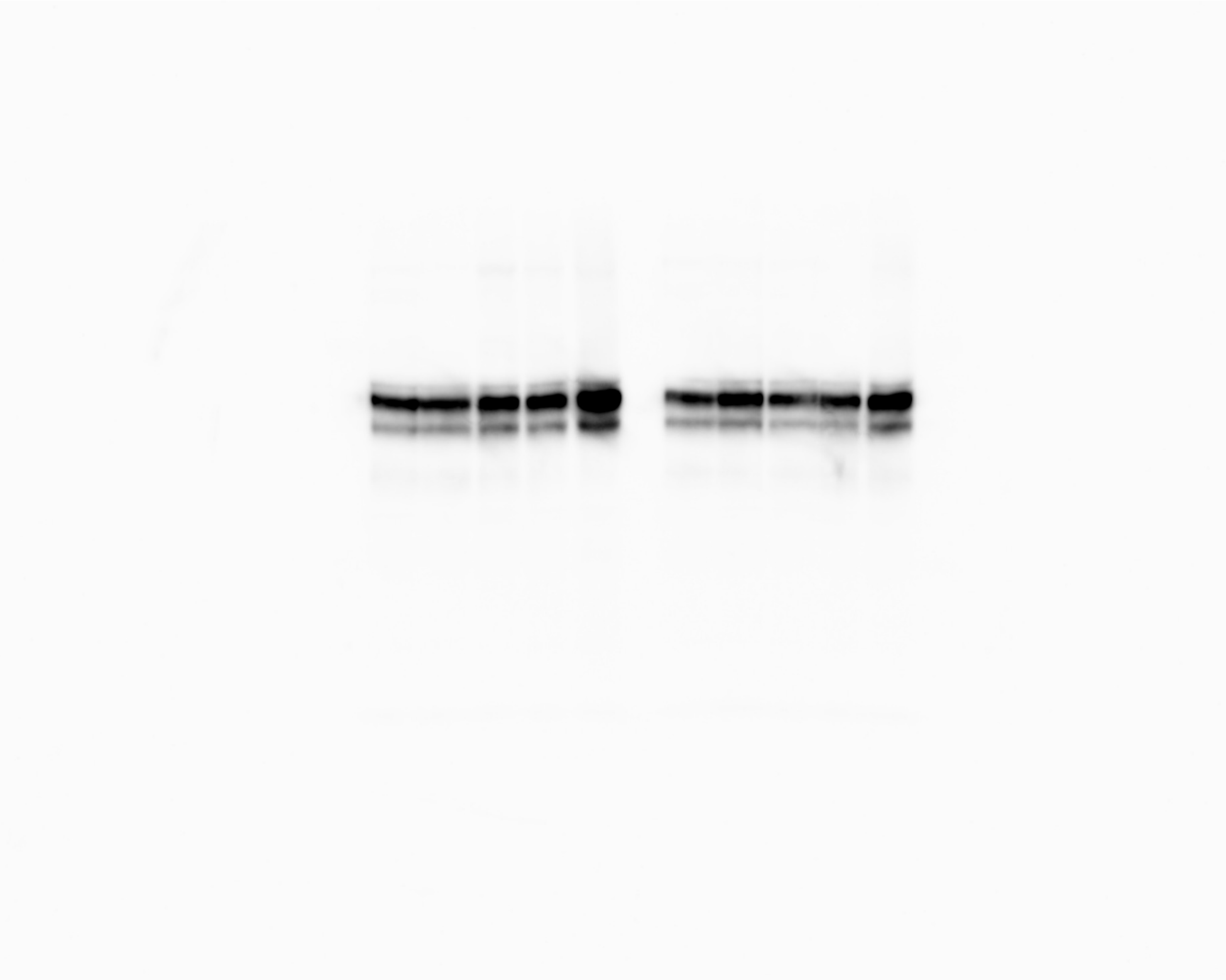

Gel image for Figure S1A (upper panel) and S1B (upper panel)

(A) WT mock pP3-4G pP15-4G pP10-4G

(B) WT mock cP2-4G pP64-4G pP35-4G

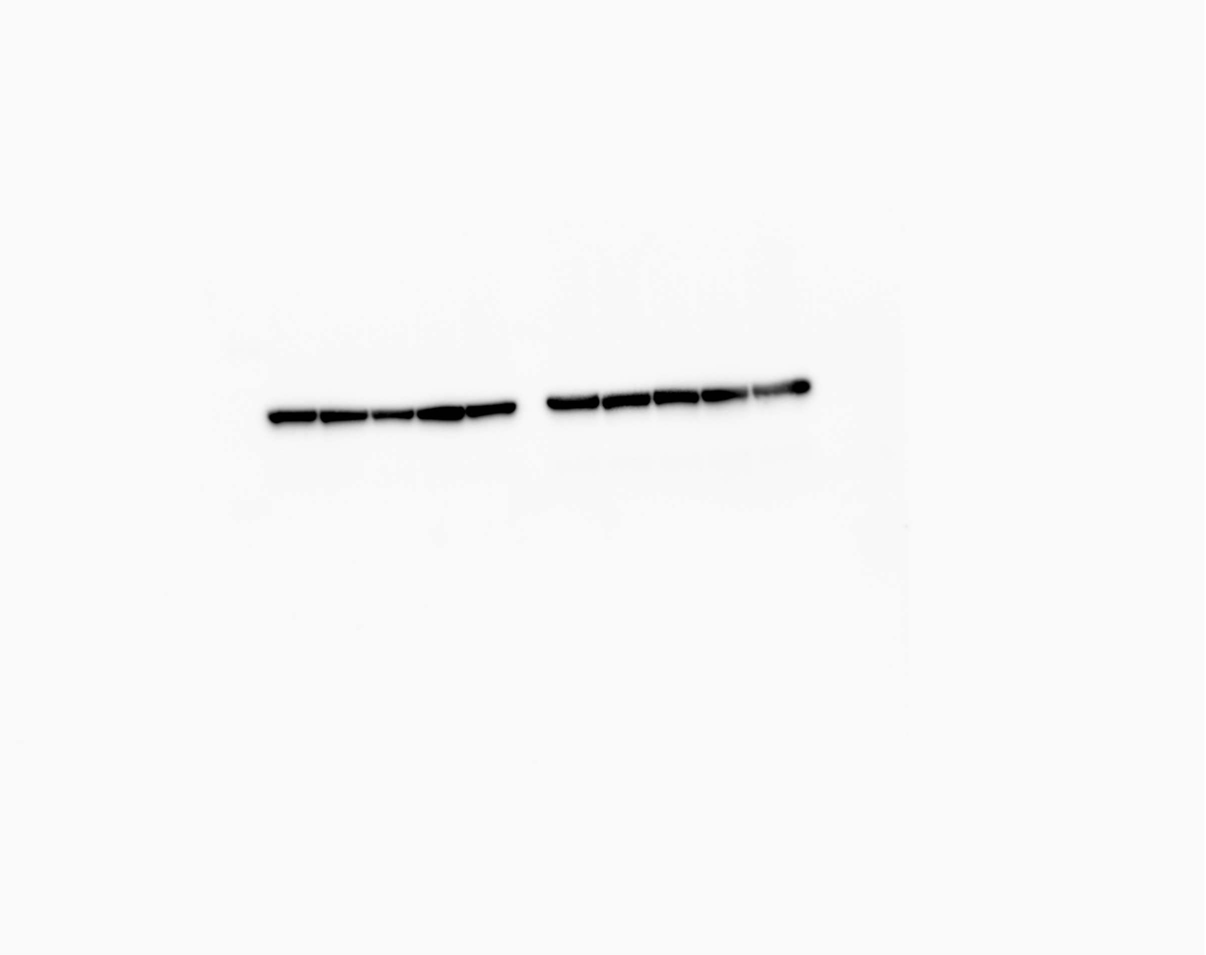

Gel image for Figure S1A (lower panel) and S1B (lower panel)

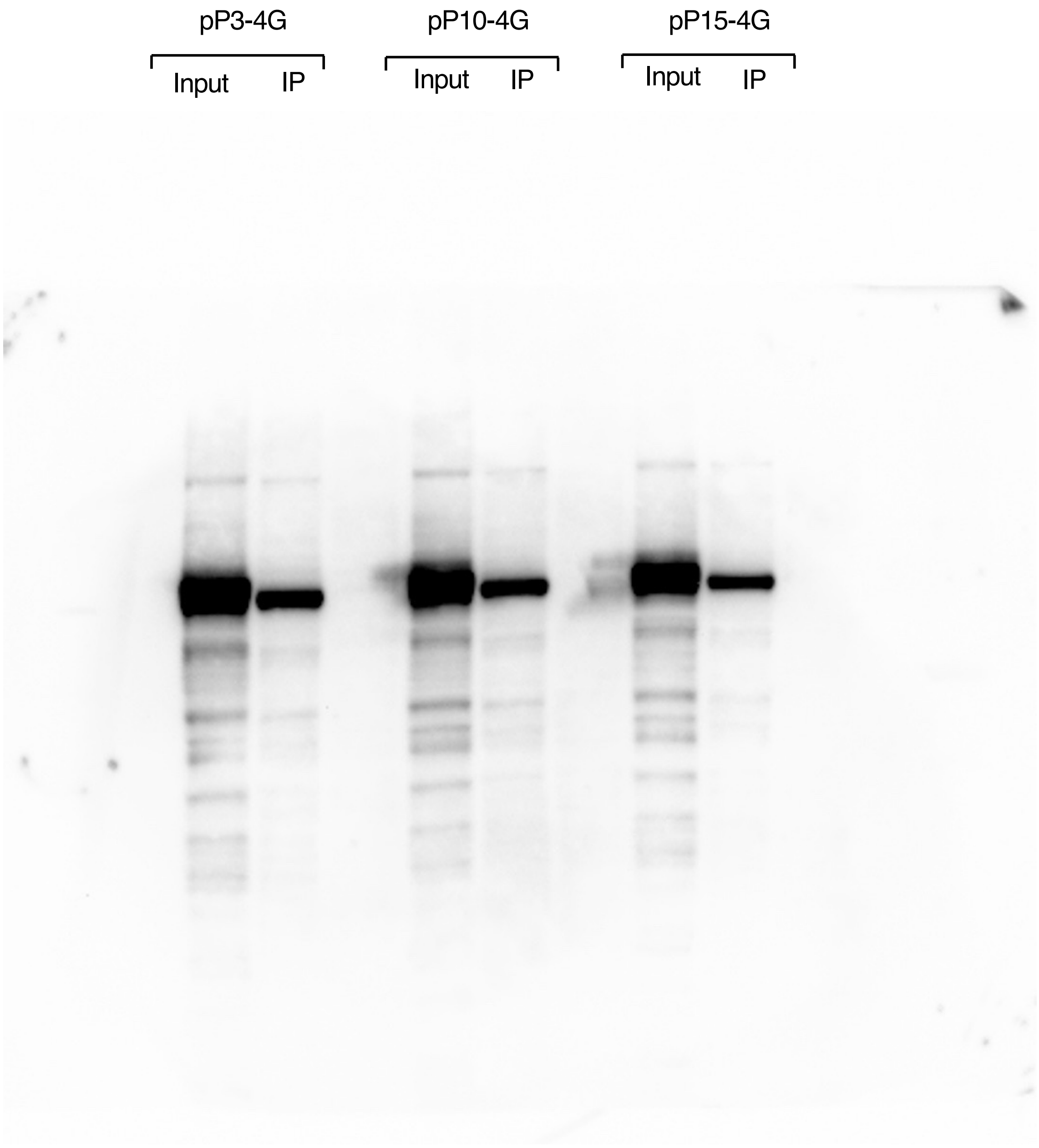

Gel image for Figure S2A

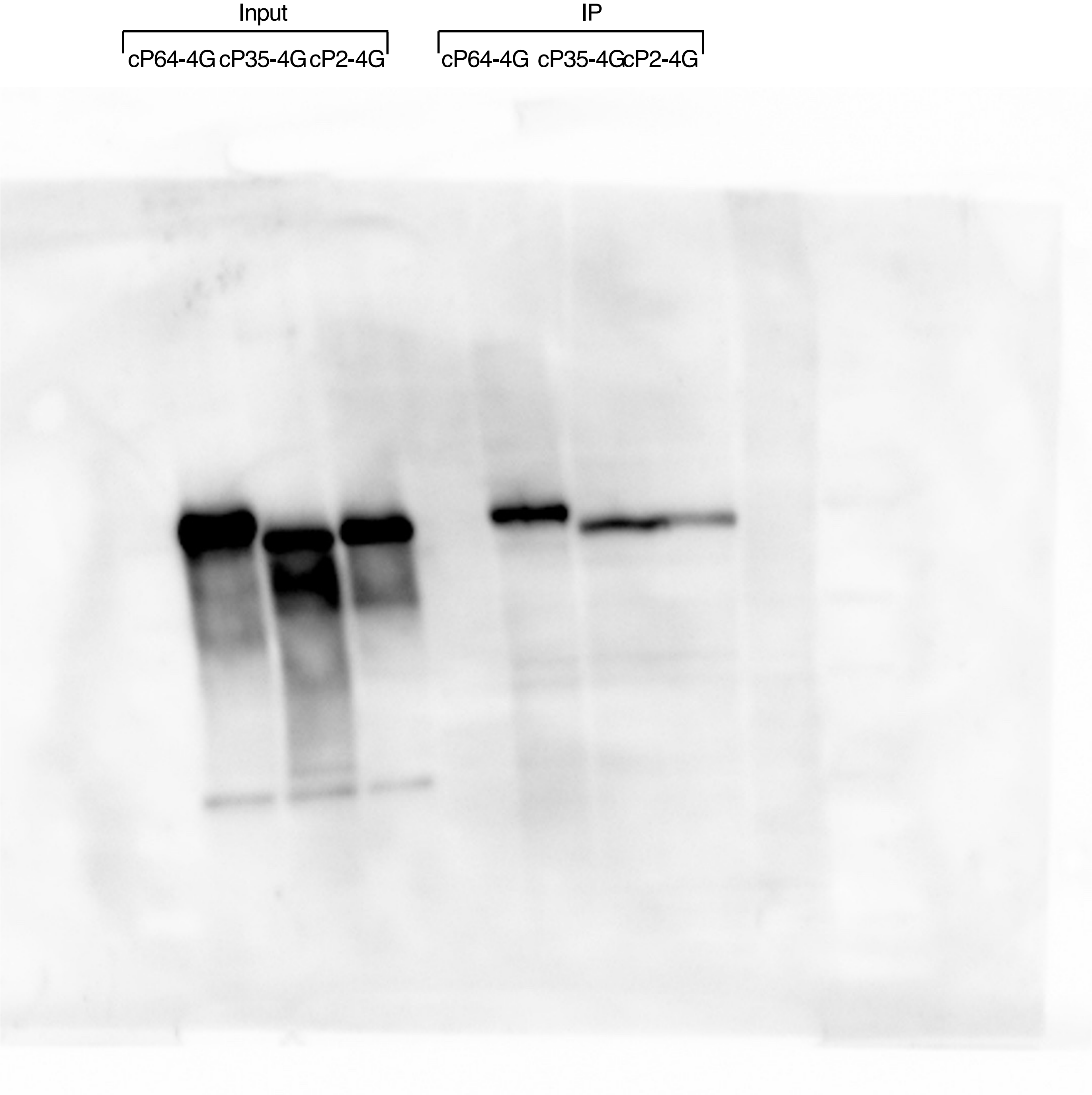

Gel image for Figure S2B

ori m1 m2 m3 m4 t1 t2 t3 t4 t5 t6

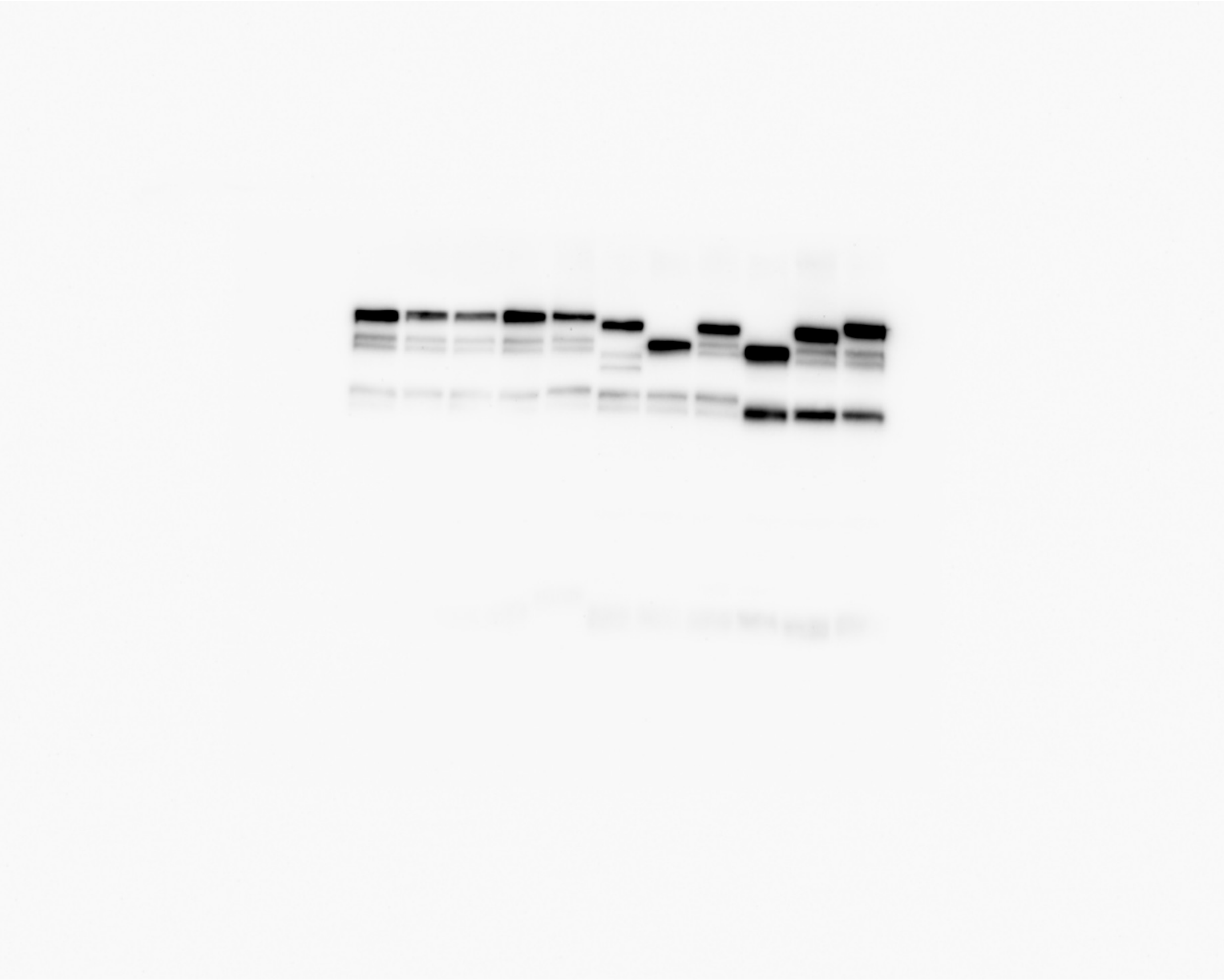

Gel image for Figure S4
